# Computational modeling of stent failure during crimping and deployment in coronary arteries

**DOI:** 10.1007/s10237-026-02091-5

**Published:** 2026-06-20

**Authors:** Alexandros Tragoudas, Gerhard A. Holzapfel, Fadi Aldakheel

**Affiliations:** 1 Institute of Mechanics and Computational Mechanics, Leibniz Universität Hannover, Hannover, Germany; 2 Institute of Biomechanics, Graz University of Technology, Graz, Austria; 3 Department of Structural Engineering, Norwegian University of Science and Technology (NTNU), Trondheim, Norway

**Keywords:** Cardiovascular disorder, Stent, Crimping and deployment, Failure mechanism, Phase-field, Large-deformation, Coronary artery, Anisotropy, Open-access codes

## Abstract

Crimping and deployment of coronary stents involve severe finite deformations, multibody contact, and complex loading-unloading sequences that critically influence their structural integrity and long-term performance. This study presents a 3D phase-field fracture framework for simulating the onset and evolution of metal stent failure during crimping and balloon-assisted deployment in coronary arteries modeled as an anisotropic, hyperelastic material. The proposed framework combines finite-strain elastoplasticity with a phase-field description of ductile fracture, implemented as a dedicated user element (UEL) in Abaqus and validated against experimental stress–strain data for stainless steel stents to accurately capture plastic deformation, damage initiation, and softening. In parallel, a second UEL is developed for the arterial wall, incorporating anisotropic hyperelasticity to represent the layered mechanical response of intima, media, and adventitia. Fully coupled simulations of the stent-balloon-artery system reproduce the complete crimp-hold-release and expansion sequence, explicitly capturing contact interactions, stress localization at crowns and connectors, and progressive damage accumulation under realistic physiological conditions. The simulations reveal that fracture is initiated already during the crimping phase and continues to evolve during balloon expansion, resulting in localized damage zones, residual stresses, and elastic recoil after balloon deflation. Comparative analyses of representative stent designs (e.g., open-cell and closed-cell configurations with varying strut thickness and geometry) demonstrate how design features, loading paths, and arterial anisotropy govern damage evolution, failure progression, and post-deployment mechanical performance. The proposed model establishes a robust computational framework for failure-aware evaluation of coronary stents under finite strains, providing new insights for optimizing stent design and deployment strategies. The corresponding source code in this study is openly available at https://doi.org/10.25835/666phabc to support further research.

## Introduction

Cardiovascular diseases, particularly atherosclerosis, remain the leading cause of mortality worldwide (Holzapfel et al. 2000; Cilla et al. 2014; Di Tomaso et al. 2011; Hansson et al. 1989; Kolodgie et al. 2007; Gierig et al. 2024; Song et al. 2020; Libby et al. 2019; Stone et al. 2012; Bäck et al. 2013). To restore blood flow in narrowed arteries, angioplasty with stent implantation has become a standard medical procedure. Although highly effective, the mechanical interaction between the stent, the balloon, and the arterial wall is inherently complex, involving large deformations, nonlinear material behavior, contact interactions, and anisotropic tissue response. These mechanical processes strongly influence stent integrity, arterial injury, elastic recoil, and long-term clinical outcome. Consequently, understanding and predicting the biomechanical mechanisms governing angioplasty and stent deployment remains essential and continues to motivate advanced computational modeling and simulation studies.

Over the past two decades, finite element (FE) modeling has become a central tool for investigating the mechanical behavior of cardiovascular stents during crimping, deployment, and *in vivo* service. Early numerical studies primarily focused on stent expansion and arterial stress distributions, demonstrating the strong influence of stent geometry, strut thickness, and cell topology on lumen gain and stent-induced vessel injury (Migliavacca et al. 2002; Lally et al. 2005). Subsequent works extended these approaches to patient-specific geometries reconstructed from medical imaging, enabling more realistic assessments of stent-artery interaction and vessel straightening (Auricchio et al. 2011, 2012; Kiousis et al. 2007). Parameterized cross-sectional models of the atherosclerotic artery were developed to investigate the influence of plaque components on the stress distribution during balloon angioplasty, thereby improving biomechanical insights and risk assessment (Kwakman et al. 2025). Significant progress has also been achieved in constitutive modeling of arterial tissue. Fiber-reinforced hyperelastic formulations, particularly those based on the Holzapfel-Ogden framework, have become the standard for capturing the anisotropic, nonlinear response of arterial layers under finite strains (Holzapfel and Ogden 2010). These models have been successfully employed in simulations of stent deployment to quantify arterial stresses and identify injury-related mechanical indicators (Holzapfel et al. 2005; Mortier et al. 2010). Parallel efforts have addressed mechanobiological aspects such as in-stent restenosis by coupling arterial mechanics with growth and remodeling models (Fereidoonnezhad et al. 2017).

With respect to stent design, extensive numerical studies have investigated the role of geometry and material selection, including comparisons between open-cell and closed-cell configurations, metallic versus polymeric stents, and different connector layouts (Mani et al. 2007; Schiavone et al. 2017). These works clearly demonstrate that design features govern stress concentrations, recoil, and arterial loading. More recent studies have emphasized that realistic modeling of balloon folding, pleating, and stent crimping is essential for reliable design assessment, as crimping-induced residual stresses are carried over into deployment and significantly influence stent expansion behavior and stress distributions (Geith et al. 2019).

Despite these advances, most existing computational studies treat stent integrity implicitly, assuming the stent material to remain intact throughout crimping and deployment. Stent failure has predominantly been investigated in the context of long-term fatigue under cyclic loading, using continuum damage mechanics or fracture-mechanics-based approaches (Argente dos Santos et al. 2012; Auricchio et al. 2016). While these studies provide valuable insight into durability, they do not address damage initiation during the deployment process itself. Experimental and clinical evidence, however, indicate that stents are subjected to severe plastic deformation and high local stresses already during crimping and expansion, and that deployment failures and strut damage are not uncommon in practice (Cantor et al. 1998; Zhao et al. 2013). However, a unified computational framework capable of explicitly capturing the initiation and evolution of fractures in stents during the entire crimping-deployment sequence under finite strains, while consistently taking into account realistic stent geometries, contact interactions and arterial anisotropy, is still lacking.

Motivated by these limitations, this work presents a finite-strain phase-field-based computational framework for modeling fracture evolution in coronary stents during crimping and deployment, with open-access source codes. The proposed approach combines finite-strain elastoplasticity with a phase-field description of ductile fracture, enabling the continuous representation of damage initiation, propagation, and localization without prescribing crack paths. The model is implemented as a dedicated user element in Abaqus and validated against experimental stress–strain data for stainless steel (Ambati et al. 2016), a material widely used in balloon-expandable stents. Full three-dimensional (3D) simulations reproduce the entire sequence of crimping, holding, releasing and balloon expansion, explicitly resolving the contact interactions between stent, balloon, and arterial wall. Several representative stent geometries inspired by industrial designs are investigated, allowing a systematic assessment of how design features such as cell topology and strut dimensions influence stress localization and failure evolution. To capture a realistic arterial response, the coronary artery is modeled as a three-layer composite structure representing the intima, media, and adventitia, and is determined by an anisotropic hyperelastic formulation based on Holzapfel’s theory (Holzapfel et al. 2005). This enables the investigation of the coupled effects of stent deformation, arterial anisotropy, and contact-induced loading on damage development, residual stresses, and elastic recoil after balloon deflation.

The structure of this paper is organized as follows. Section 2 introduces the biomechanical setting of angioplasty with stent implantation and outlines the modeling assumptions. Section 3 presents the formulations for hyperelasticity, plasticity and nonlinear isotropic hardening at finite strains, together with their coupling to the phase-field fracture framework, transverse anisotropy and the corresponding numerical implementation of the computational models. Section 4 discusses the numerical results, focusing on damage evolution during crimping and deployment, design-dependent failure mechanisms, and post-deployment behavior. Finally, Sect. 5 summarizes the main findings, limitations, and perspectives for future developments.

## Biomechanical framework

In all living organisms, complex chemical reactions and biological processes continuously occur to ensure proper functionality. In the human body, the core of these biological processes lies in the interactions between various cell types. These cells require nutrients to stay alive, which are delivered through the blood transported in the arteries. Arteries play a crucial role in the human body and differ in both function and mechanical characteristics. One common way to categorize them is by size, distinguishing between large, medium, and small vessels. Medium-sized arteries, known as coronary arteries, supply the heart with nutrients via the blood flow (Milutinović et al. 2020).

**Fig. 1:**
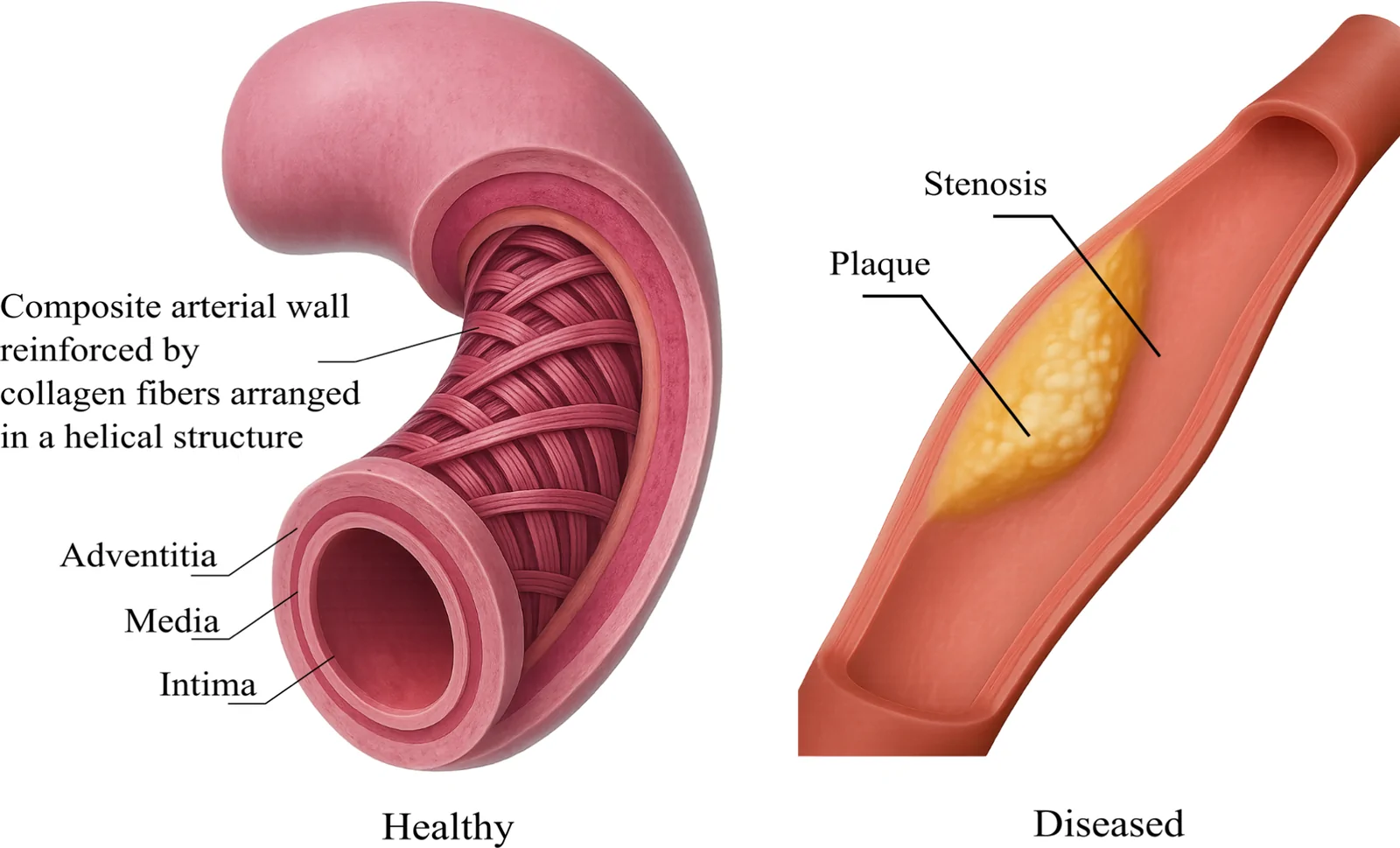
Comparison of a healthy artery and a stenosed artery, highlighting the layered arterial structure and plaque formation

The artery is composed of three main layers, as shown in Fig. 1. These layers, from the innermost to the outermost, are referred to as the intima, media, and adventitia (Holzapfel et al. 2000). Each of these layers possesses distinct mechanical and biological properties. Each layer consists of a non-collagenous matrix, which introduces an isotropic material response, and two families of collagen fibers helically wound along the longitudinal axis with different orientation angles in each layer. In most studies, a symmetric arrangement of these fibers is assumed. However, recent research suggests accounting for collagen fiber dispersion (Holzapfel et al. 2019). The presence of these fibers induces an anisotropic mechanical response, giving each layer orthotropic behavior. Therefore, a constitutive theory for fiber-reinforced solids must be employed to properly model the arterial mechanical response. Due to this composition, arteries are commonly regarded as composite materials, consisting of a matrix reinforced by fibers.

The intima is the innermost layer of the artery. In a healthy young muscular artery, the intima is very thin and makes an insignificant contribution to the solid mechanical properties of the arterial wall. Consequently, in many studies, the artery is modeled as a two-layered tube. However, it should be noted that with age, the intima thickens, resulting in increased stiffness and a more significant mechanical contribution. Additionally, according to the standard theory of atherosclerosis formation, the intima layer plays a crucial role, neglecting it therefore leads to inaccurate results. The media is the middle layer of the artery and contains helically distributed fibers. Due to the small pitch of these fibers, they are typically aligned in the circumferential direction. This structure provides the media with high resilience and the ability to absorb loads in both the longitudinal and circumferential directions. Because of these properties, the media is the mechanically most important layer in a healthy artery. The adventitia, the outermost arterial layer, makes a substantial mechanical contribution to the stability and strength of the blood vessel. Compared to the media, the adventitia exhibits lower stiffness under load-free conditions and at low internal blood pressures. Under higher loads, however, the collagen fibers straighten, leading to increased stiffness of the adventitia. This mechanism prevents overstretching and rupturing of the artery.

*In vivo*, soft tissues exhibit a pre-stretched configuration under internal pressure. However, as in the work of Holzapfel et al. (2000), upon which the model implemented in this study is based, an opened-up, unstressed configuration is assumed. Residual stresses in the axial direction should also be considered, but for modeling simplicity, they are neglected in this work. In future studies, the authors intend to include the pre-stretch effect, following the approach of Gierig et al. (2024). The resulting stress-free reference configuration is an open-cut circular cylindrical tube, consistent with experimental studies (Holzapfel et al. 2000, 2005, 2004), which forms the basis for the subsequent constitutive description. Healthy arteries subjected to high pressures exhibit a highly nonlinear stress–strain response, characterized by a typical exponential stiffening effect. Under tension, the helically wound collagen fibers become activated, leading to structural stiffening and contributing to the characteristic anisotropic mechanical behavior. Typically, the collagen fibers are modeled with preferred direction vectors defined in the reference configuration. From these, structural tensors are computed, introducing the anisotropic contribution into the strain-energy function. Such mechanically driven arterial responses are closely linked to vascular pathologies.

One of the major causes of death worldwide is cardiovascular disease, specifically the phenomenon of atherosclerosis. This disorder develops only in medium- or large-sized arteries, such as the coronary arteries (Parton et al. 2016). Several theories and studies have been published aiming to describe the origin of atherosclerotic disease and the processes leading to plaque formation. To this day, the most widely accepted theory suggests that atherosclerosis develops as a response to injury (Milutinović et al. 2020). Nevertheless, a more recent perspective on the initiation of atherosclerotic formation has emerged, known as the outside-in theory (Gierig et al. 2024; Soleimani et al. 2023).

**Fig. 2:**
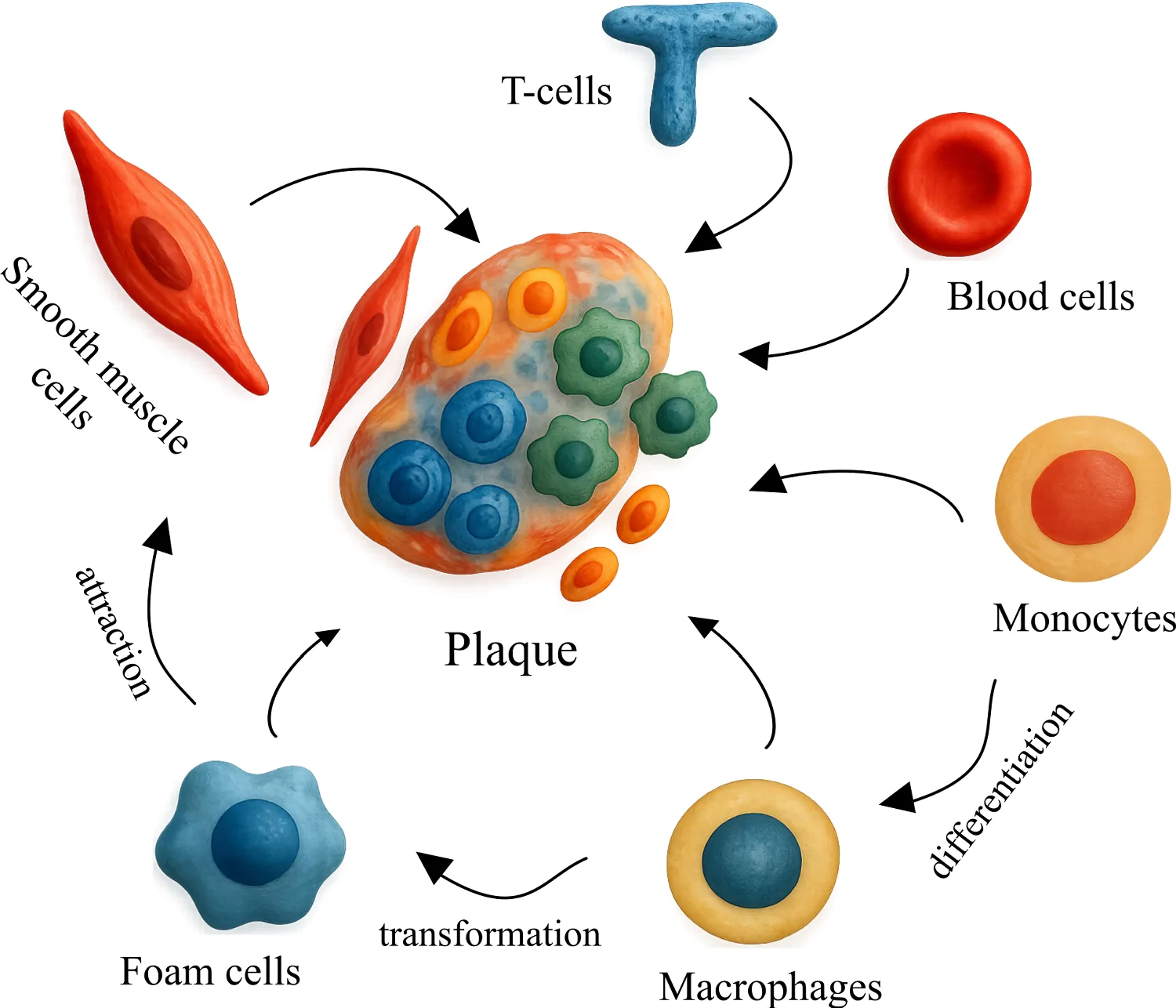
Schematic illustration of the cellular processes involved in atherosclerotic plaque formation

From a mechanical perspective, atherosclerosis leads to a hardening of blood vessels due to calcification and the loss of elasticity (Cilla et al. 2014), thereby altering the mechanical response of all arterial layers. Furthermore, plaque formation leads to a narrowing of the vessel lumen. According to most theories, plaque development is associated with the accumulation of lipids and cells in the outer regions of the intima, near the media, see Fig. 2. The main danger of stenosis lies in the significant increase in blood flow velocity, which can lead to the rupture of an unstable plaque. A plaque rupture may lead to the formation of a blood clot, which can travel through the bloodstream. Such a clot may block blood flow and potentially trigger a heart attack or stroke.

To counteract the negative effects of severe stenosis, balloon angioplasty with stent implantation has become state of the art due to its minimally invasive nature. Typically, the diameter of healthy coronary arteries ranges between 2 and 4 $\text {mm}$. In order to fit a stent into a stenosed artery, it must first be reduced in diameter, a process known as crimping. This involves compressing the stent onto a catheter along with a deflated balloon. In the literature, the crimper is often modeled as a rigid component of a thick metal tube, applying a radially controlled displacement load (Qiu et al. 2018). During crimping, the stent diameter decreases significantly, leading to residual stresses. Although the residual stresses associated with stent expansion have been extensively studied, their role in damage initiation remains largely unexplored. The computational study by Qiu et al. (2018) analyzed several stent designs and simulated crimping from an initial diameter of 3.2 $\text {mm}$ to a deformed state of 2 $\text {mm}$. The analysis revealed that the stent design significantly influences its stiffness and deformation behavior. All stents exhibited the highest stress levels at critical locations such as the crowns.

**Fig. 3:**
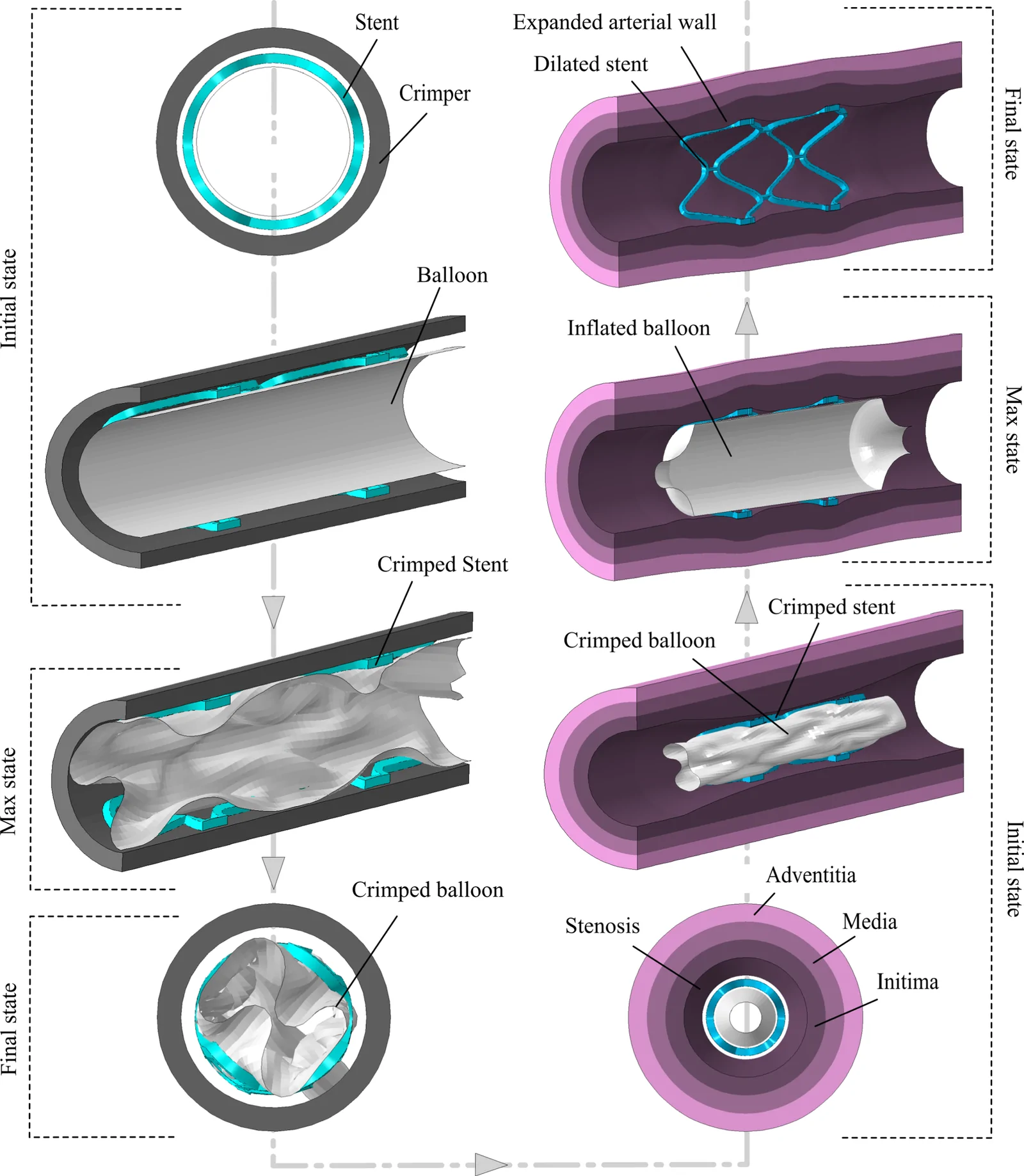
Crimping and deployment phases of the stent implantation procedure, from balloon-stent crimping to final expansion within a stenosed artery

The stent implantation procedure begins by inserting the crimped stent into the stenosed artery using a catheter. Once positioned correctly, air pressure is applied to inflate the balloon. The inflation causes radial expansion of the stent until it is fully deployed, thereby widening the artery. To help readers to better understand the procedure, Fig. 3 provides an explanation of the simulation cases used in this work. Due to the complex anatomical structure of arteries, expansion can be asymmetrically, with the temporarily adopting a characteristic dog-bone shape until maximum pressure is reached. The entire process typically takes about 1 $\text {sec}$ and consists of three phases: inflation, a short period of constant pressure, and deflation. In the final deflation stage, the stent elastically recoils, adopting its final, expanded configuration. The degree of recoil depends on the material properties and stent design. This stage also influences the final stress state in both the stent and the arterial wall, making it critical for long-term mechanical performance and fatigue resistance.

**Fig. 4:**
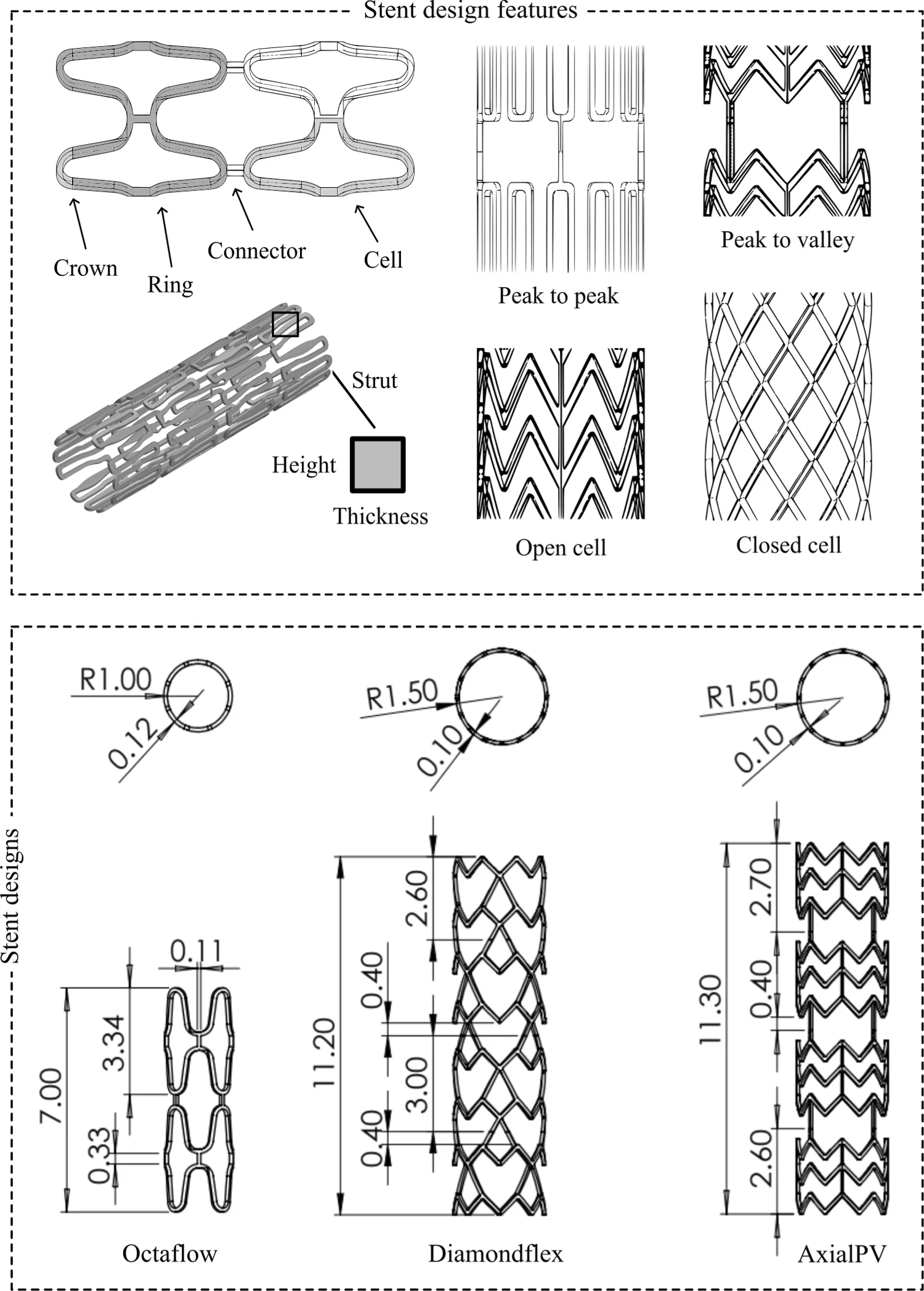
Stent geometrical features, connection configurations and corresponding technical designs in [mm]

Given recent technological advances, a significant area of stent research focuses on the influence of the geometric features of the design on overall structural and mechanical performance. These may refer to the size or shape of their features, and consequently, to the overall design (Ray et al. 2025). Stents are primarily composed of rings and connectors, which link the rings together. The rings themselves consist of struts and crowns. Important aspects that have been thoroughly investigated include how the thickness, width, and length of the struts, the width and height of the connectors, the radius of the crowns, and the overall shape of these components influence the mechanical response (Blair et al. 2019; Pant et al. 2011; Amirjani et al. 2014). The influence of these features are evaluated based on key performance indices (KPIs). These KPIs are usually divided into structural, hemodynamic and multiphysics as they are presented and discussed in detail in the work of Kapoor et al. (2024). Beyond the mechanical dimensions of these features, the arrangement of these components leads to different families of stent designs. A classical distinction is between stents with closed-cell and open-cell structures. In the latter, the rings are connected only at a few regions, which provides greater design flexibility, allowing the stent to better conform to arteries with high curvature, reducing arterial injury during deployment, and decreasing neointimal response. In contrast, closed-cell stents exhibit higher mechanical integrity, enabling them to better withstand cyclic cardiac loading. The current industry trend focuses primarily on open-cell designs (Kapoor et al. 2024; Okereke et al. 2021). Furthermore, apart from the number of connectors between rings, the type of connection serves as another categorization criterion. These are most commonly known as peak-to-peak or peak-to-valley configurations, which are also referred to as out-of-phase and in-phase, respectively. In addition to these two main categories, there is also a mid-strut connector configuration. Figure 4 illustrates the different stent design families previously introduced. Since both of these aspects need to be evaluated throughout the various phases that stents undergo after manufacturing, longitudinal (Ragkousis et al. 2014), torsional (Everett et al. 2016), and bending tests (Wang et al. 2018) are commonly performed to compare different material characteristics and design features. Additionally, the crimping process, which is required to insert the stent into the narrowed coronary artery, followed by its deployment by balloon inflation or self-expansion (Gierig et al. 2025), as well as fatigue analysis to assess lifetime prediction under physiological conditions, have all been important areas of research in recent years.

To investigate these various cases, numerical simulations have proven to be a valuable tool. In the work of Blair et al. (2019), the influence of design features was investigated, while several other studies have focused on the crimping and deployment processes (Qiu et al. 2018; De Beule et al. 2008; Okereke et al. 2021, 2023), also considering the effects of the balloon (Djukic et al. 2019; Schiavone and Zhao 2015; Schiavone et al. 2014, 2013; Mortier et al. 2010). An important aspect of stent technology concerns the post-deployment behavior. Therefore, simulating the fatigue response has been a crucial research focus (Auricchio et al. 2014, 2015; Guerchais et al. 2016; dos Santos et al. 2012; Harewood and McHugh 2007), with many studies comparing their results to experimental data reported in the literature (Auricchio et al. 2016; Donnelly et al. 2017; Agarwal et al. 2007; Wiersma et al. 2006).

Another important aspect that is the subject of research in the stent industry concerns the manufacturing material itself. Currently, there are three main categories: non-biodegradable metals, biodegradable metals, and bioabsorbable, polymer-based materials (Pogorielov et al. 2017; Jiang et al. 2017; Auras et al. 2010; McMahon et al. 2018; Mani et al. 2007). In this work, the authors chose stainless steel (316L SS) as it is one of the most commonly used materials for manufacturing stents, and extensive literature data are available to validate the results. They investigate the structural response of several stent designs illustrated in Fig. 4 during the crimping process, as well as during deployment in coronary arteries under both normal physiological and extreme conditions.

## Constitutive material modeling

To accurately capture the interplay between irreversible plastic deformation and progressive fracture, a coupled framework integrating finite plasticity theory with phase-field modeling was developed. This approach enables the simulation of ductile failure mechanisms under large strains, where both material hardening and crack evolution significantly influence the structural response. This section provides a brief introduction to the kinematics of finite plasticity as presented in Simo and Hughes (2006); Simo and Ju (1989); Simo (1988a, 1988b), together with the foundations of phase-field theory (Miehe et al. 2016; Aldakheel et al. 2018).

### Basic kinematics

Compared to the small-strain theory, where an additive decomposition of the total strain tensor into elastic and plastic parts $\boldsymbol{\varepsilon } = \boldsymbol{\varepsilon }_{\textrm{e}} + \boldsymbol{\varepsilon }_{\textrm{p}}$ is used, the finite-strain theory employs a multiplicative decomposition of the deformation gradient ${\textbf {F}} = {\textbf {F}}^{\textrm{e}} {\textbf {F}}^{\textrm{p}}$ with the second-order tensors ${\textbf {F}}^{\textrm{e}}$ and ${\textbf {F}}^{\textrm{p}}$ corresponding to the elastic and plastic deformation gradients, respectively. Following classical continuum mechanics theory, the Green-Lagrange strain tensors ${\textbf {E}}$ and ${\textbf {E}}^{\textrm{p}}$ are calculated in terms of the right Cauchy–Green tensors ${\textbf {C}}$ and ${\textbf {C}}^{\textrm{p}}$ as

1 $$\begin{aligned}&{\textbf {E}} = \frac{1}{2}({\textbf {C}} - {\textbf {I}}) = \frac{1}{2}({\textbf {F}}^{\text {T}} {\textbf {F}} - {\textbf {I}}) \quad \text {and} \nonumber \\&{\textbf {E}}^{\textrm{p}} = \frac{1}{2}({\textbf {C}}^{\textrm{p}} - {\textbf {I}}) = \frac{1}{2}({\textbf {F}}^{\textrm{p}^{\text {T}}} {\textbf {F}}^{\textrm{p}} - {\textbf {I}}) \end{aligned}$$

where ${\textbf {I}}$ denotes the second-order identity tensor. In continuum mechanics, two main configurations exist, the initial and the current configuration. The concept of finite plasticity, as thoroughly introduced by Simo and Hughes (2006), is defined in the current configuration. Therefore, it is important to introduce the Euler-Almansi strain tensors as

2 $$\begin{aligned}&{\textbf {e}} = \frac{1}{2}({\textbf {I}} - {\textbf {b}}^{-1}) \quad \text {and} \quad {\textbf {e}}^{\textrm{e}} = \frac{1}{2}({\textbf {I}} - {\textbf {b}}^{\textrm{e}^{-1}}) \quad \text {with}\nonumber \\&{\textbf {b}} = {\textbf {F}} {\textbf {F}}^{\text {T}} \text {,} \quad {\textbf {b}}^{\textrm{e}} = {\textbf {F}}^{\textrm{e}} {\textbf {F}}^{\textrm{e}^{\text {T}}}= {\textbf {F}} {\textbf {C}}^{\textrm{p}^{\text {-1}}} {\textbf {F}}^{\text {T}} \end{aligned}$$

in terms of the elastic left Cauchy–Green tensor ${\textbf {b}}^{\textrm{e}}$ and the plastic right Cauchy–Green tensor ${\textbf {C}}^{\textrm{p}}$. Various plasticity and hardening formulations have been introduced in the literature for yield criteria. This work follows the classical $J_{2}$ plasticity model with isotropic hardening, as it effectively describes the behavior of metallic materials. Therefore, the von Mises yield criterion is presented as

3 $$\begin{aligned} &  f(\boldsymbol{\tau }_{\text {dev}},\alpha ) = \Vert \boldsymbol{\tau }_{\text {dev}}\Vert - \sqrt{\frac{2}{3}}R(\alpha ) \le 0 \quad \text {with} \nonumber \\ &  R(\alpha ) = \sigma _{\text {Y}} + (\sigma _{\infty } - \sigma _{\text {Y}})\left( 1 - \exp (-\delta \alpha )\right) + H\alpha \end{aligned}$$

where $\boldsymbol{\tau }_{\text {dev}}$ refers to the deviatoric part of the Kirchhoff stress tensor, while R(α) corresponds to the chosen exponential hardening function. The internal hardening variable α is also referred to as the equivalent plastic strain. The scalar quantities $\sigma _{\text {Y}}$, $\sigma _{\infty }$, δ and *H* denote the material constants for the yield stress, ultimate tensile stress, saturation parameter, and isotropic hardening parameter, respectively. Due to the nonlinear characteristics of the hardening function, a local Newton–Raphson procedure is required to solve the local residual and obtain the incremental plastic multiplier $\Delta \gamma$.

In the upcoming subsection, a detailed description of how this residual is formulated and solved will be presented. In order to update the history variables ${\textbf {C}}^{\textrm{p}}$ and α, evolution equations must be introduced as

4 $$\begin{aligned}&\dot{{\textbf {C}}}^{\mathrm {p-1}} = -\frac{2}{3}\Delta \gamma \,\text {tr}({\textbf {b}}^{\textrm{e}}) \, {\textbf {F}}^{-1} {\textbf {n}} {\textbf {F}}^{-\text {T}} \nonumber \\&\text {with} \hspace{2cm} \dot{\alpha } = \sqrt{\frac{2}{3}}\Delta \gamma \end{aligned}$$

where $\text {tr}({\textbf {b}}^{\textrm{e}})$, ${\textbf {n}} = \boldsymbol{\tau }_{\text {dev}} / \Vert \boldsymbol{\tau }_{\text {dev}} \Vert$, and $\Delta \gamma$ denote the first invariant of the elastic left Cauchy–Green tensor, the stress direction vector, and the plastic Lagrange multiplier, respectively. To ensure thermodynamic consistency, the Karush–Kuhn–Tucker (KKT) conditions must be satisfied at each iteration step, i.e.

5 $$\begin{aligned} \Delta \gamma&\ge 0,&f(\boldsymbol{\tau }_{\text {dev}}, \alpha )&\le 0,&\Delta \gamma f(\boldsymbol{\tau }_{\text {dev}}, \alpha )&= 0 \end{aligned}$$

For the phase-field problem, a sharp-crack surface topology $\Gamma \rightarrow \Gamma _l$ is regularized by the crack surface functional as

6 $$\begin{aligned} \Gamma _l(d) = \int _{\Omega _{\text {Stent}}} \gamma _l(d,\nabla d)\,\textrm{d}V \quad \text {with} \quad \gamma _l(d,\nabla d) = \frac{1}{2l_c} d^2 + \frac{l_c}{2} \Vert \nabla d\Vert ^2 \end{aligned}$$

which is in line with Aldakheel (2016), where $\Omega _{\text {Stent}}$ denotes the reference volume of the stent. It is based on the crack surface density function $\gamma _l$ per unit volume of the solid and the fracture length scale parameter $l_c$ that governs the regularization. To describe a purely geometric approach to phase-field fracture, the regularized crack phase-field d∈[0,1] is obtained by a minimization principle of diffusive crack topology

7 $$\begin{aligned} d = \text {Arg} \inf _{d} \Gamma _l(d) \quad \text {with} \quad d = 1 \;\text {on}\; \Gamma \subset \Omega _{\text {Stent}} \end{aligned}$$

yielding the Euler equation $d - l_c^2 \Delta d = 0$ in $\Omega _{\text {Stent}}$ along with the Neumann-type boundary condition $\nabla d \cdot \boldsymbol{N} = 0$ on $\partial \Omega _{\text {Stent}}$. Here, $\boldsymbol{N}$ is the outward unit normal vector on the surface. A value of d=0 corresponds to an undamaged region, whereas d=1 represents a fully fractured state.

### Material modeling of stent

The principle underlying this work lies in the minimization of a pseudo-potential, commonly referred to as the total energy potential Π. The constitutive material modeling of stent is expressed as the sum of the elastic $\Psi ^{\textrm{e}}$, plastic $\Psi ^{\textrm{p}}$, and fracture energy contributions $\Psi ^{\textrm{f}}$ as

8 $$\begin{aligned} \Pi (\bar{{\textbf {b}}}^{\textrm{e}}, J, \alpha , d)= &  \int _{\Omega _{\text {Stent}}} \Big [ \Psi ^{\textrm{e}} (\bar{{\textbf {b}}}^{\textrm{e}}, J, d) + \Psi ^{\text {p}} (\alpha ) + \Psi ^{\textrm{f}} (d) \Big ] \textrm{d}V \nonumber \\= &  \int _{\Omega _{\text {Stent}}} \Big [ g(d) \Big ( \Psi ^{\textrm{e}} (\bar{{\textbf {b}}}^{\textrm{e}}, J)+ \Psi ^{\text {p}} (\alpha ) \Big ) + \Psi ^{\textrm{f}}(d, \nabla d) \Big ] \textrm{d}V \end{aligned}$$

The elastic energy function $\Psi ^{\textrm{e}}$ is decomposed into volumetric $\Psi _{\text {vol}}^\textrm{e}$ and deviatoric parts $\Psi _{\text {dev}}^\textrm{e}$ as

9 $$\begin{aligned} &  \Psi = \Psi ^{\text {e}} (\bar{{\textbf {b}}}^{\text {e}}, J) = \Psi _{\text {vol}}^\textrm{e} + \Psi _{\text {dev}}^\textrm{e} \quad \text {where} \nonumber \\ &  \Psi _{\text {vol}}^\textrm{e} = \frac{\kappa }{2} \left( \frac{(J^\textrm{e})^2 - 1}{2} - \ln J^\textrm{e} \right) \quad \text {and}\nonumber \\ &  \Psi _{\text {dev}}^\textrm{e} = \frac{\mu }{2} \left( \text {tr}[\bar{{\textbf {b}}}^\textrm{e}] - 3 \right) \end{aligned}$$

The deviatoric part of the elastic left Cauchy–Green tensor is defined as $\bar{{\textbf {b}}}^\textrm{e} = J^{\textrm{e}^{-2/3}} {\textbf {b}}^\textrm{e}$, while the volumetric part depends on the determinant of the elastic deformation gradient, $J^{\text {e}} = J = \det {\textbf {F}}$ for isochoric plastic flow. The parameters μ and κ correspond to the shear and bulk moduli of the material, respectively. Based on Miehe et al. (2015, 2010), the elastic free-energy function is decomposed into positive and negative parts as

10 $$\begin{aligned} \Psi ^\text {e} (\bar{{\textbf {b}}}^\text {e}, J, d) = g(d) \Psi ^\text {e}_+ (\bar{{\textbf {b}}}^\text {e}, J) + \Psi ^\text {e}_- (J) \end{aligned}$$

together with the corresponding volumetric and deviatoric components

11 $$\begin{aligned} &  J < 1 \quad {\left\{ \begin{array}{ll} \Psi ^\text {e}_+ = \Psi ^\text {e}_{\text {dev}} (\bar{{\textbf {b}}}^\text {e}) \\ \Psi ^\text {e}_- = \Psi ^\text {e}_{\text {vol}} (J) \end{array}\right. } \nonumber \\ &  \text {and} \quad J \ge 1 \quad {\left\{ \begin{array}{ll} \Psi ^\text {e}_+ = \Psi ^\text {e}_{\text {dev}} (\bar{{\textbf {b}}}^\text {e}) + \Psi ^\text {e}_{\text {vol}} (J) \\ \Psi ^\text {e}_- = 0 \end{array}\right. } \end{aligned}$$

For the degradation function *g*(*d*), a quadratic form is considered (Miehe et al. 2016; Sidharth and Rao 2023) as

12 $$\begin{aligned} g(d) = (1-d)^{2}, \hspace{1cm} g(0) = 1, \hspace{1cm} g(1) = 0 \end{aligned}$$

Following Simo (1988a), Simo (1988b) and Dittmann et al. (2018), the plastic energy contribution is defined in terms of the internal variable α as

13 $$\begin{aligned} &  \Psi (\alpha ) = \int _{0}^{\alpha } R(\alpha ) \ \textrm{d}\alpha = \int _{0}^{\alpha } \left[ \sigma _{\text {Y}} + (\sigma _{\infty } - \sigma _{\text {Y}})\right. \nonumber \\ &  \left. \left( 1 - \exp (-\delta \alpha )\right) + H\alpha \right] \textrm{d}\alpha \end{aligned}$$

The fracture contribution is modeled using phase-field theory as Miehe et al. (2015); Aldakheel et al. (2018)

14 $$\begin{aligned} \Psi ^f(d, \nabla d) = \big [ 1 - g(d) \big ] \psi _c + 2 \frac{\psi _c}{\zeta } l_c \gamma _l(d, \nabla d) \end{aligned}$$

where, $\psi _c > 0$ is a critical fracture energy and ζ controls the post-critical range after crack initiation.

#### Time integration scheme

The subscripts $(\cdot )_{n}$ and $(\cdot )_{n+1}$ refer to the values of a variable at the previous time step $t_{n}$ and the current time step $t_{n+1}$, respectively. Hereby, a theory for the coupling of plasticity with the phase-field modeling of fracture is introduced. Thus, the trial stress tensor is multiplied by the phase-field degradation function, in a manner similar to that introduced in Ambati et al. (2016); Borden et al. (2016); Kumar and Patel (2025), to incorporate the phase-field formulation as

15 $$\begin{aligned}&\boldsymbol{\tau }_{\text {dev},n+1}^{\text {tr}} = g(d) \mu \, \text {dev} \left[ \bar{{\textbf {b}}}_{n+1}^{\text {e},\text {tr}} \right] \quad \text {with} \ \ \ \bar{{\textbf {b}}}_{n+1}^{\text {e},\text {tr}} = \bar{{\textbf {F}}}_{n+1} {\textbf {C}}_n^{\text {p-1}} \bar{{\textbf {F}}}_{\text {n+1}}^\textrm{T} \ \ \nonumber \\&\text {where} \ \ \bar{{\textbf {F}}}_{n+1} = J_{n+1}^{-1/3} {\textbf {F}}_{n+1} \ \ \text {and} \ \ J_{n+1} = \det [{\textbf {F}}_{n+1}] \end{aligned}$$

The history variables are initialized as $\alpha _{n} = 0$ and ${\textbf {C}}_n^{\text {p}} = {\textbf {I}}$. The superscript $\Box ^\text {tr}$ refers to the trial state (lies within the elastic region). This initialization allows the necessary computations of the trial yield function as

16 $$\begin{aligned} f_{n+1}^{\text {tr}}= &  \Vert \boldsymbol{\tau }_{\text {dev},n+1}^{\text {tr}} \Vert - \sqrt{\frac{2}{3}} g(d) \ R(\alpha _n) \le 0 \quad \text {with}\nonumber \\ R(\alpha _{n})= &  \sigma _{\text {Y}} + (\sigma _{\infty } - \sigma _{\text {Y}})\left( 1 - \exp (-\delta \alpha _{n})\right) + H\alpha _{n} \end{aligned}$$

For an elastic loading step, no update of the history variables is required, i.e., ${\textbf {C}}_{n+1}^{\text {p}} = {\textbf {C}}_{n}^{\text {p}}$ and $\alpha _{n+1} = \alpha _{n}$. On the other hand, if the state lies in the plastic region, the predictor-corrector algorithm is activated to compute the Lagrange multiplier $\Delta \gamma$ and the history variables are updated in the following algorithms (Box 1–2).

**Figure:**
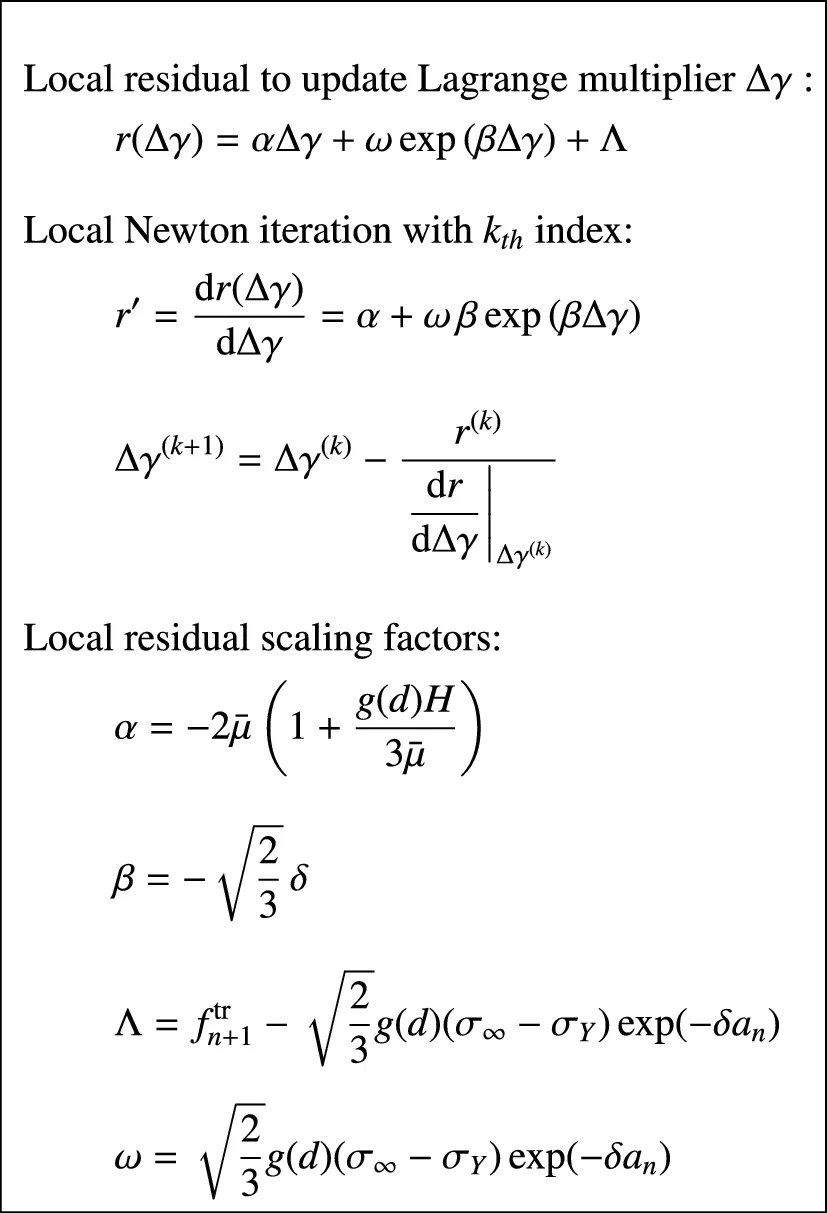
**Box 1**: Local residual Newton iteration scheme

**Figure:**
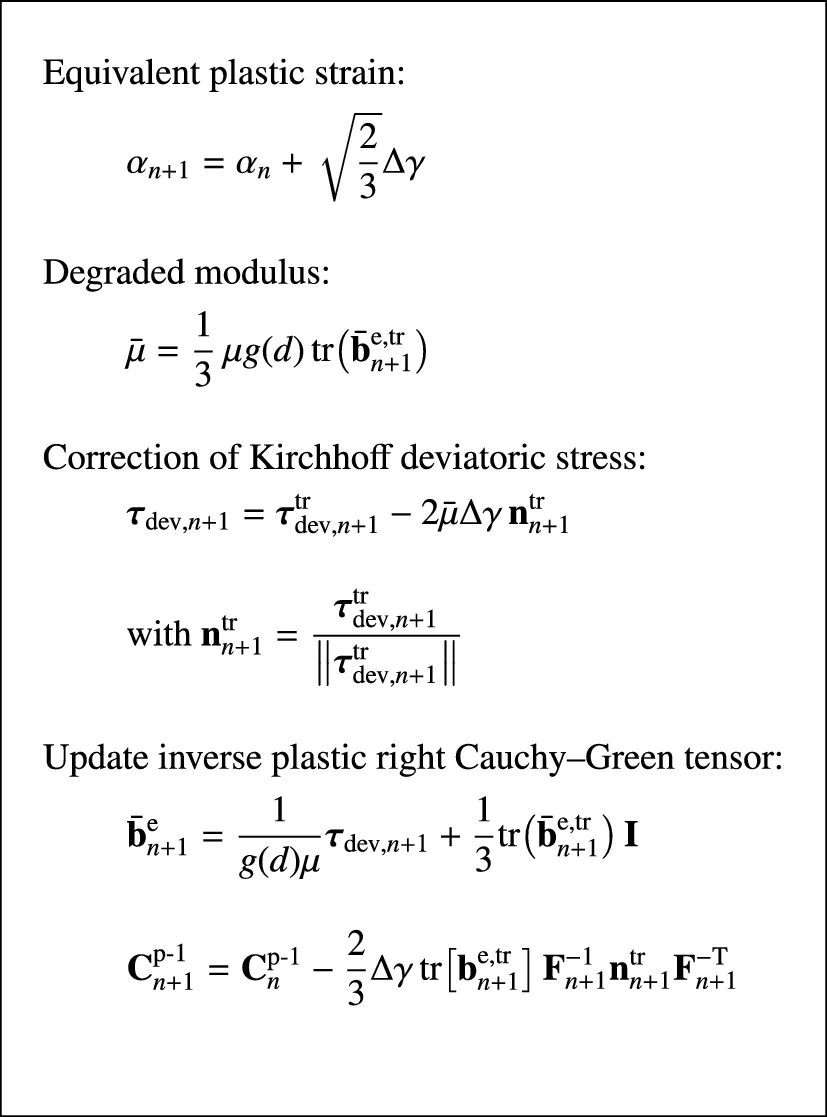
**Box 2**: Updates of internal variables

#### Stress and tangent moduli

Following the Coleman-Noll procedure, the Cauchy stress tensor $\boldsymbol{\sigma }$ is obtained as

17 $$\begin{aligned} &  \boldsymbol{\sigma } = \frac{2}{J} {\textbf {b}}^\textrm{e} \left( \frac{\partial \Psi ^\textrm{e}}{\partial {\textbf {b}}^\textrm{e}} \right) = \frac{2}{J} {\textbf {b}}^\textrm{e} \left( \frac{\partial \Psi _{\text {vol}}^\textrm{e}}{\partial {\textbf {b}}^\textrm{e}} + \frac{\partial \Psi _{\text {dev}}^\textrm{e}}{\partial {\textbf {b}}^\textrm{e}} \right) \nonumber \\ &  = \frac{1}{J} \left\{ \frac{\kappa }{2} \left( J^2 - 1 \right) {\textbf {I}} + \mu \operatorname {dev}[\bar{{\textbf {b}}}^\textrm{e}] \right\} = \frac{1}{J} ( \boldsymbol{\tau }_{\text {vol}} + \boldsymbol{\tau }_{\text {dev}}) \end{aligned}$$

where the tensor $\boldsymbol{\tau }_{\text {vol}}$ corresponds to the volumetric part of the Kirchhoff stress tensor $\boldsymbol{\tau }$. Under the assumption that the volumetric part of the strain energy is not degraded during compression but is activated only under tension, the Cauchy stress tensor $\boldsymbol{\sigma }$ takes on the form

18 $$\begin{aligned}&\boldsymbol{\sigma } = \frac{2}{J} \textbf{b}^\textrm{e} \left( g(d)\frac{\partial \Psi _{+}^\textrm{e}}{\partial \textbf{b}^\textrm{e}} + \frac{\partial \Psi _{-}^\textrm{e}}{\partial \textbf{b}^\textrm{e}} \right) \nonumber \\&\quad \text {with} \quad \boldsymbol{\sigma } = {\left\{ \begin{array}{ll} \displaystyle \frac{1}{J} \Big ( g(d)\boldsymbol{\tau }_{\text {dev}} + \boldsymbol{\tau }_{\text {vol}} \Big ), & \text {if } J < 1 \\ \displaystyle \frac{1}{J}\, g(d) \left( \boldsymbol{\tau }_{\text {dev}} + \boldsymbol{\tau }_{\text {vol}} \right) , & \text {if } J \ge 1 \end{array}\right. } \end{aligned}$$

Analogously, the second derivative of the strain-energy function with respect to the elastic left Cauchy–Green tensor yields the elastic spatial material tangent tensor

19 $$\begin{aligned} \boldsymbol{\mathbbm {c}}_{n+1}^{\text {e}, \text {tr}} = \frac{1}{J} \left[ \boldsymbol{\mathbbm {c}}_{\text {vol}, n+1}^{\text {e}, \text {tr}} + \boldsymbol{\mathbbm {c}}_{\text {dev}, n+1}^{\text {e}, \text {tr}} \right] \end{aligned}$$

Similarly to the stress tensor decomposition, the degradation of the volumetric part $\boldsymbol{\mathbbm {c}}_{\text {vol}}$ occurs only under tension

20 $$\begin{aligned} \boldsymbol{\mathbbm {c}}_{\text {vol}, n+1}^{\text {e}, \text {tr}} = {\left\{ \begin{array}{ll} \kappa \left[ J^2 {\textbf {I}} \otimes {\textbf {I}} - (J^2 - 1) {\mathbb {I}} \right] & \text {if } J < 1 \\ g(d) \ \kappa \left[ J^2 {\textbf {I}} \otimes {\textbf {I}} - (J^2 - 1) {\mathbb {I}} \right] & \text {if } J \ge 1 \end{array}\right. } \end{aligned}$$

where $\mathbb {I}$ denotes the fourth-order identity tensor. While the consistent elastic deviatoric part is formulated as

21 $$\begin{aligned} \boldsymbol{\mathbbm {c}}_{\text {dev}, n+1}^{\text {e}, \text {tr}}= &  g(d) \frac{2}{3} \mu \text {tr}(\bar{{\textbf {b}}}^{\text {e}}) \left( \mathbb {I} - \frac{1}{3} {\textbf {I}} \otimes {\textbf {I}} \right) - \frac{2}{3} \left[ \left( \boldsymbol{\tau }_{\text {dev}, n+1}^\text {tr} \otimes {\textbf {I}} \right) \right. \nonumber \\ &  \left. + \left( {\textbf {I}} \otimes \boldsymbol{\tau }_{\text {dev}, n+1}^\text {tr} \right) \right] \end{aligned}$$

In Simo and Hughes (2006), a consistent representation of the spatial elastoplastic material tangent moduli is provided, as formulated subsequently. The numerical factors to be computed in the case of plasticity are introduced, where $R^{\prime }$ denotes the first derivative of the hardening function *R* with respect to the internal variable *a*. Depending on the material behavior, the hardening function can take different forms. In the simplest case of linear isotropic hardening $R^{\prime }=H$.

**Figure:**
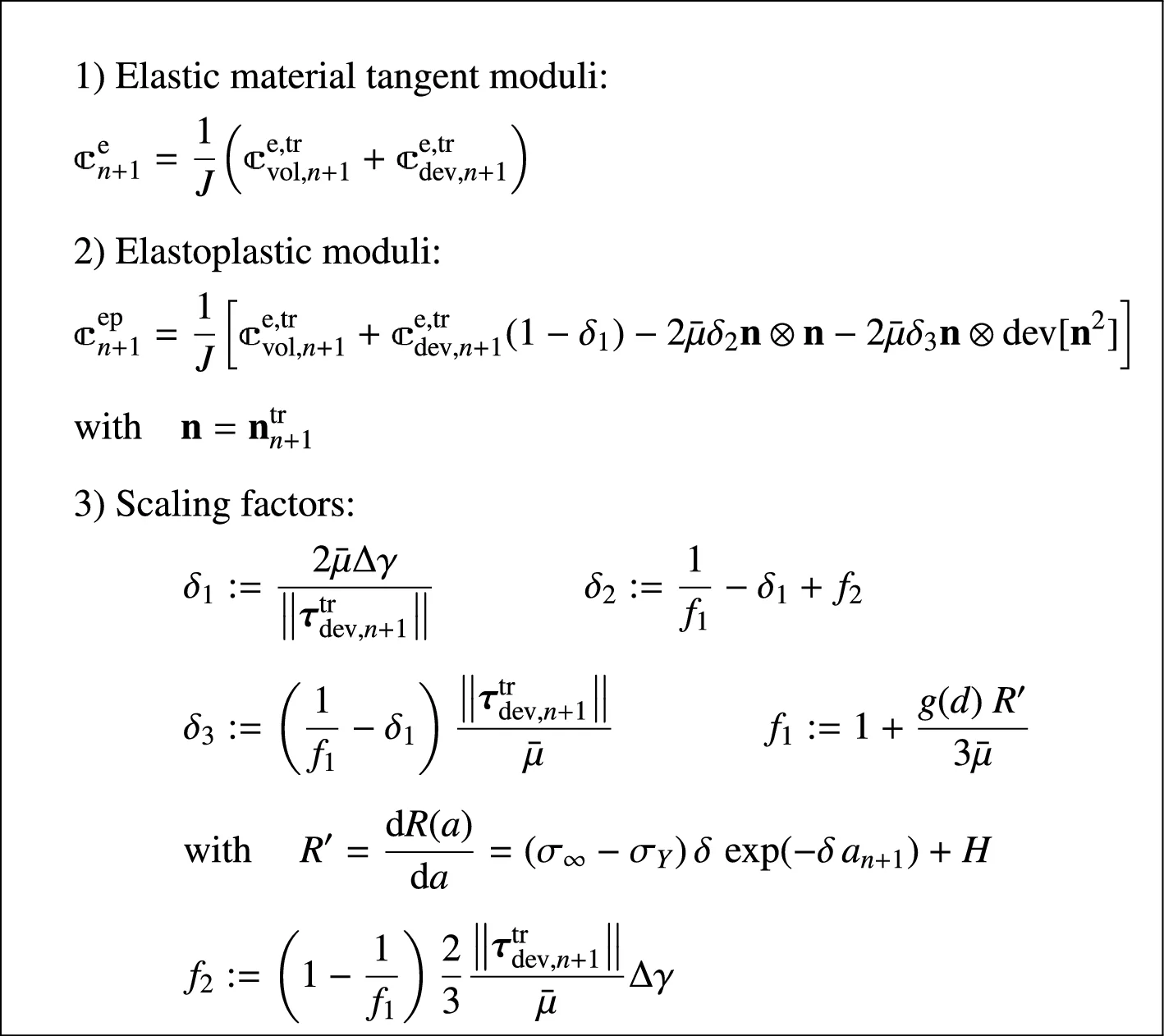
**Box 3**: Elastoplastic material tangent, scaling factors and nonlinear hardening derivative

### Material modeling of coronary arteries

So far, the previous subsections have introduced a material modeling formulation that accounts for finite elastoplasticity combined with the phase-field method to accurately describe the physical behavior of metallic stents. In addition, to more precisely capture the real structural response of stents during deployment, a second material framework is implemented to account for the anisotropic behavior of the coronary arteries. Thus,

23 $$\begin{aligned} \Psi = \Psi _{\text {vol}} + \Psi _{\text {dev}} + \Psi _{\text {aniso}} \end{aligned}$$

Compared to the stent material model, in this case the free-energy function is formulated with respect to the initial configuration, using a volumetric deviatoric split (Holzapfel et al. 2000; Nagaraja et al. 2021; Gierig et al. 2024; Soleimani et al. 2023) to account for the high incompressibility effects

24 $$\begin{aligned} &  \Psi _{\text {vol}} = \frac{\kappa }{4} \Big (J^{2} - 1 - 2 \ln J \Big ) \quad \text {and} \nonumber \\ &  \Psi _{\text {dev}} = \frac{\mu }{2} \Big ( J^{-2/3} I_1 - 3 \Big ) \quad \text {with} I_1 = \text {tr}[{\textbf {C}}] = {\textbf {F}}: {\textbf {F}} \end{aligned}$$

The anisotropic behavior of the coronary arteries is taken into account using an exponential formulation, as introduced in Holzapfel et al. (2005). This formulation was adopted, motivated by the comprehensive experimental characterization of coronary arteries reported in Holzapfel et al. (2005), including the mechanical properties of the arterial layers intima, media, and adventitia, along with the corresponding constitutive parameters κ>0, μ>0, $k_1 > 0$, $k_2 > 0$, and $\rho \in [0,1]$, which allowed the authors to properly calibrate their arterial material model as

25 $$\begin{aligned} \Psi _{\text {aniso}} = \frac{k_{1}}{k_{2}} \sum _{i=1}^{n} \Bigg ( e^{ \big ( k_{2} (1 - \rho )(I_1 -3)^2 + k_{2} \rho (I_4^i-1)^2 \big )} - 1 \Bigg ) \end{aligned}$$

Following the approach of Gültekin et al. (2016), the incompressibility of the soft tissue is ensured by considering a bulk modulus $\kappa \approx 10^3 \mu$ relative to the shear modulus, resulting in a Poisson’s ratio $\nu \approx 0.5$ and thus a highly incompressible response. As reported in the literature, coronary arteries exhibit an anisotropic mechanical response. The fourth invariant, $I_{4}^i$ captures this anisotropic effect by incorporating the structural tensor **M** and it is only activated when $I_{4}^i > 1$ (depending on the direction vector ${\textbf {a}}$) as

26 $$\begin{aligned} I_{4}^i = \text {tr}\big [{\textbf {C}} \cdot {\textbf {M}}_i\big ] \quad \text {with} \quad {\textbf {M}}_i = {\textbf {a}}_i \otimes {\textbf {a}}_i \end{aligned}$$

Each layer of the coronary artery contains two families of fibers. The index *n* in Eq. (25) refers to the number of fiber families in each layer and the corresponding anisotropic energy that contributes to the total energy response. The formulation of the direction vector depends on whether a flat or cylindrical specimen is considered in the simulation. For a cylindrical tube, the fibers can be visualized as helical spirals, and the direction vectors for the first and second fiber families are described as

27 $$\begin{aligned} \textbf{a}_{1} = \begin{pmatrix} -\cos (\theta _{el}) \sin (\theta _{az}) \\ \cos (\theta _{el}) \cos (\theta _{az}) \\ \sin (\theta _{el}) \end{pmatrix}, \quad \textbf{a}_{2} = \begin{pmatrix} \cos (\theta _{el}) \sin (\theta _{az}) \\ -\cos (\theta _{el}) \cos (\theta _{az}) \\ \sin (\theta _{el}) \end{pmatrix} \end{aligned}$$

The elevation angle $\theta _{el}$ is a parameter, while the azimuth angle $\theta _{az}$ is derived from the initial material coordinates. The corresponding stress and material tangent tensors are composed of volumetric, deviatoric, and anisotropic components, i.e.

28 $$\begin{aligned} &  {\textbf {S}} = 2 \frac{\partial \Psi }{\partial {\textbf {C}}}= {\textbf {S}}_{\text {vol}} + {\textbf {S}}_{\text {dev}} + \sum _i^n {\textbf {S}}_{\text {aniso,i}} \quad \text {and} \nonumber \\ &  \boldsymbol{\mathbb{C}} = 2 \frac{\partial {\textbf {S}}}{\partial {\textbf {C}}} = \boldsymbol{\mathbb{C}}_{\text {vol}} + \boldsymbol{\mathbb{C}}_{\text {dev}} + \sum _i^n \boldsymbol{\mathbb{C}}_{\text {aniso,i}} \end{aligned}$$

Derivation of the volumetric, deviatoric and anisotropic strain-energy components with respect to right Cauchy–Green tensor ${\textbf {C}}$ returns the corresponding second Piola–Kirchoff stress tensors according to

29 $$\begin{aligned} {\textbf {S}}_{\text {vol}}&= \frac{\kappa }{2} \big ( J^2 -1 \big )\textbf{C}^{-1} \quad \text {and} \quad {\textbf {S}}_{\text {dev}} = \mu \ J^{-2/3} \big ( \textbf{I} - \frac{I_{1}}{3} \,\textbf{C}^{-1} \big ) \nonumber \\ {\textbf {S}}_{\text {aniso,i}}&= \frac{2 k_1}{k_2} \nonumber \Big [ 2 k_2 (1 - \rho ) (I_{1} - 3) e^{k_2 (1 - \rho )(I_{1} - 3)^2} e^{ k_2 \rho (I_{4}^i - 1)^2 )} \ \textbf{I} \nonumber \\&\quad + e^{ k_2 (1 - \rho )(I_{1} - 3)^2 } \, 2 k_2 \rho (I_{4}^i - 1) e^{ k_2 \rho (I_{4}^i - 1)^2 } \ \textbf{M}_{i} \Big ] \end{aligned}$$

The corresponding material tangent stiffness matrices can be computed as

30 $$\begin{aligned} \mathbb{C}_{\text {vol}}&= \kappa J^2 \left[ \textbf{C}^{-1} \otimes \textbf{C}^{-1} - \Big ( 1 - \frac{1}{J^2} \Big ) \textbf{C}^{-1} \odot \textbf{C}^{-1} \ \right] \nonumber \\ \mathbb{C}_{\text {dev}}&= -\frac{2}{3}\mu J^{-2/3} \left( \textbf{C} ^{-1} \otimes \textbf{I} + \textbf{I} \otimes \textbf{C}^{-1} \right) \nonumber + \frac{2}{9}\mu J^{-2/3} {I}_{1}\left( \textbf{C}^{-1} \otimes \textbf{C}^{-1} \right) + \frac{2}{3}\mu J^{-2/3} {I}_{1}\left( \textbf{C}^{-1} \odot \textbf{C}^{-1} \right) \nonumber \\ \boldsymbol{\mathbb{C}}_{\text {aniso,i}}&= \frac{4 k_1}{k_2} \Bigg [ 2 k_2 (1 - \rho ) \Big ( e^{k_2 (1 - \rho )(I_{\text {1}} - 3)^2} (\textbf{I} \otimes \textbf{I}) \nonumber + 2 k_2 (1 - \rho )(I_{\text {1}} - 3)^2 e^{k_2 (1 - \rho )(I_{\text {1}} - 3)^2} (\textbf{I} \otimes \textbf{I}) \Big ) e^{k_2 \rho (I_{4}^i - 1)^2} \nonumber \\&\quad + \Big ( 2 k_2 (1 - \rho )(I_{\text {1}} - 3) e^{k_2 (1 - \rho )(I_{\text {1}} - 3)^2} \Big ) \nonumber \Big ( 2 k_2 \rho (I_{4}^i - 1) e^{k_2 \rho (I_{4}^i - 1)^2} \Big )\nonumber (\textbf{I} \otimes \textbf{M}_{i} + \textbf{M}_{i} \otimes \textbf{I} ) \nonumber \\&\quad + \Big ( e^{k_2 (1 - \rho )(I_{\text {1}} - 3)^2} 2 k_2 \rho \Big ) \nonumber \Big (e^{k_2 \rho (I_{4}^i - 1)^2} (\textbf{M}_{i} \otimes \textbf{M}_{i}) + (I_{4}^i - 1) 2 k_2 \rho (I_{4}^i - 1) e^{k_2 \rho (I_{4}^i - 1)^2} (\textbf{M}_{i} \otimes \textbf{M}_{i}) \Big ) \Bigg ] \end{aligned}$$

with the mathematical property $(\textbf{C})^{-1} \odot (\textbf{C})^{-1} = \left( \textbf{C}^{-1}_{\text {ik}} \textbf{C}^{-1}_{\text {jl}} + \textbf{C}^{-1}_{\text {il}} \textbf{C}^{-1}_{\text {jk}} \right) / 2$.

### Governing equations

Using the quantities introduced above, we define the governing partial differential equations of the coupled problem related to the coronary artery and stent.

**Figure:**
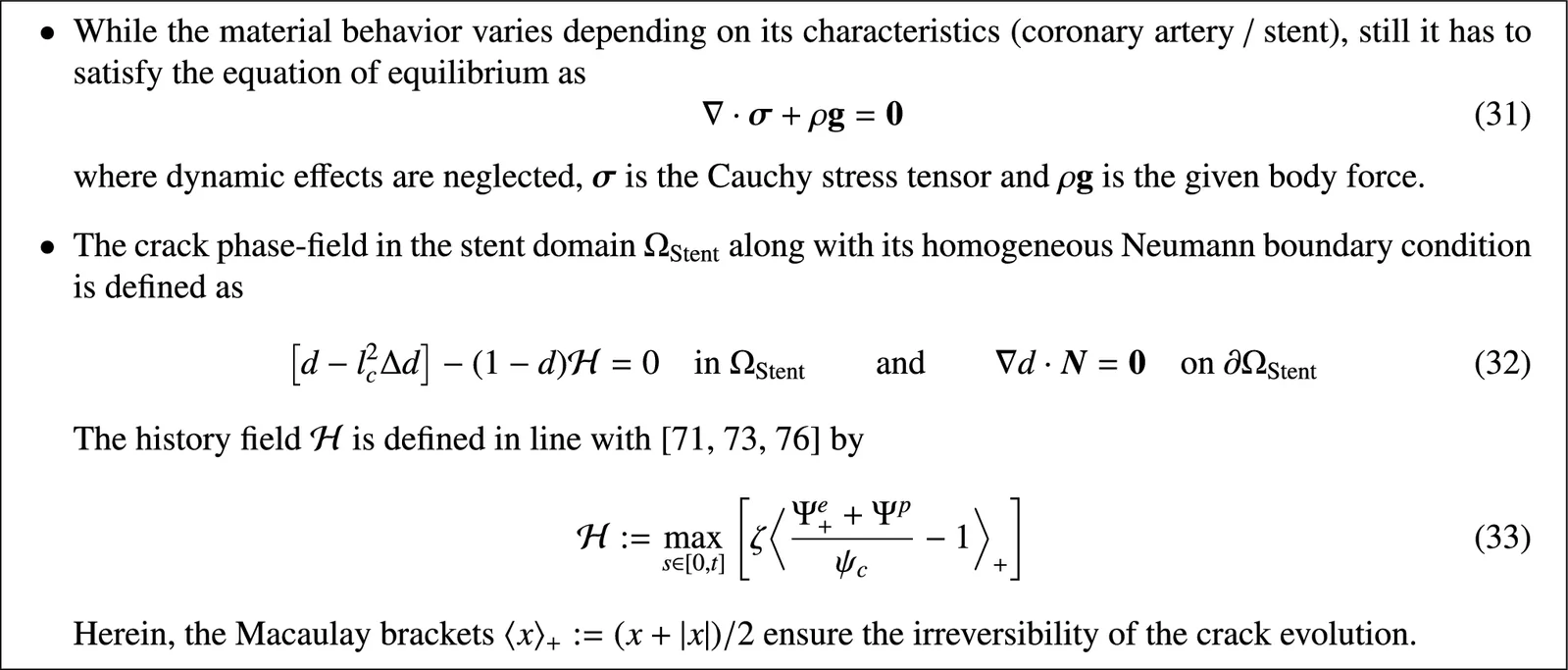

### Algorithmic interpretation of workflow

**Fig. 5:**
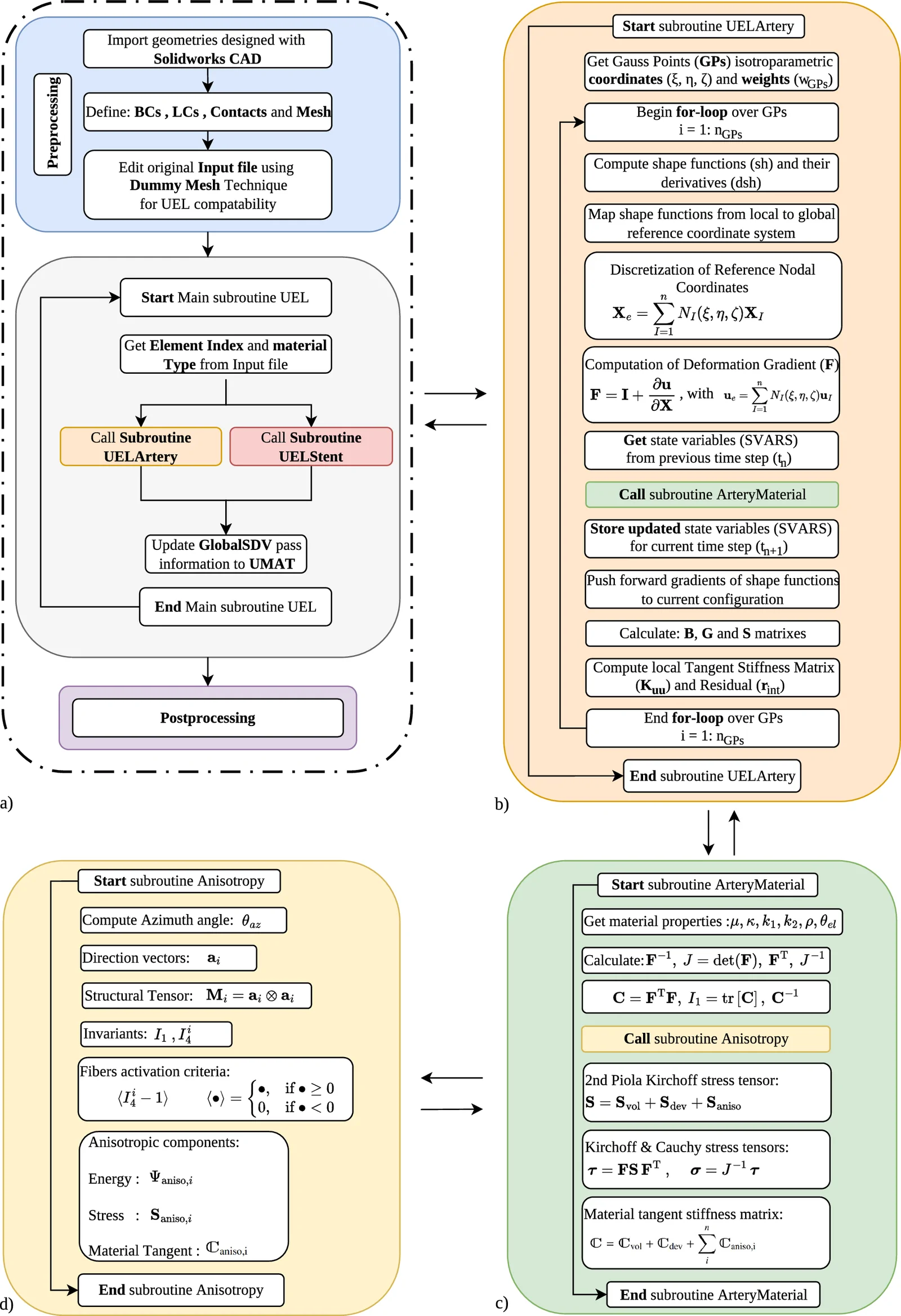
Algorithmic workflow of the UEL-based models, corresponding to the available open-access Abaqus implementation

In Fig. 5a) an illustrative description of the complete workflow of this study is presented. The process begins with creating the relevant edited input file, which contains all the information required for an executable simulation. To achieve this, the relevant geometries are first imported into Abaqus. Thereafter, the appropriate contact formulations, boundary conditions (BCs), loading conditions (LCs), and corresponding mesh refinements are defined. The original input file is then edited by implementing a dummy mesh technique, enabling the visualization of SVAR quantities computed with the UELs.

**Fig. 6:**
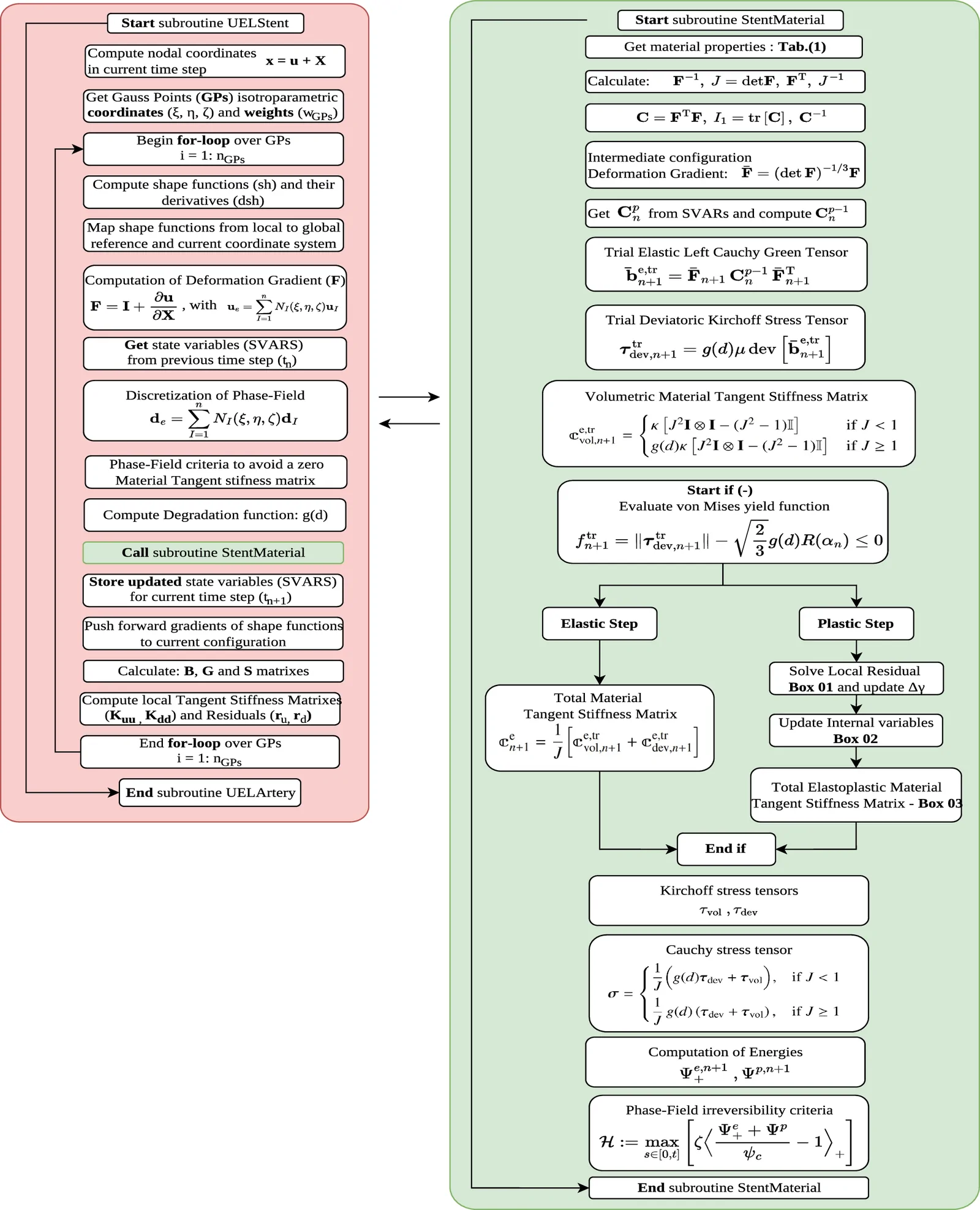
Algorithmic steps of the UEL-based stent model as implemented in the available open-access Abaqus codes

During execution, the main UEL subroutine of Abaqus is called, containing the corresponding UELs for the stent and the coronary artery. In each iteration, Abaqus provides information on the current element index and material type, allowing the relevant UEL for the current computation step to be identified. After the computation, a GlobalSDV matrix is updated, referring to a UMAT subroutine that enables the visualization of the SVAR values. Figures 5b–d and 6 provide algorithmic interpretations of how the finite element discretization and material formulation for the coronary artery and stent take place.

## Numerical results

In the previous section the mathematical and numerical models employed in this work are introduced. To ensure the reliability of the proposed models, this section presents two validation cases for the stent and the coronary artery, respectively, with comparisons to experimental data available in the literature.

Subsequently, stent-specific simulations are presented. The first case examines phase-field evolution during crimping, while the second considers stent expansion within a coronary artery, accounting for the coupled response of the stent-artery system. To demonstrate the generality of the proposed framework, three stainless steel stents with distinct design characteristics are analyzed.

### Validation

**Fig. 7:**
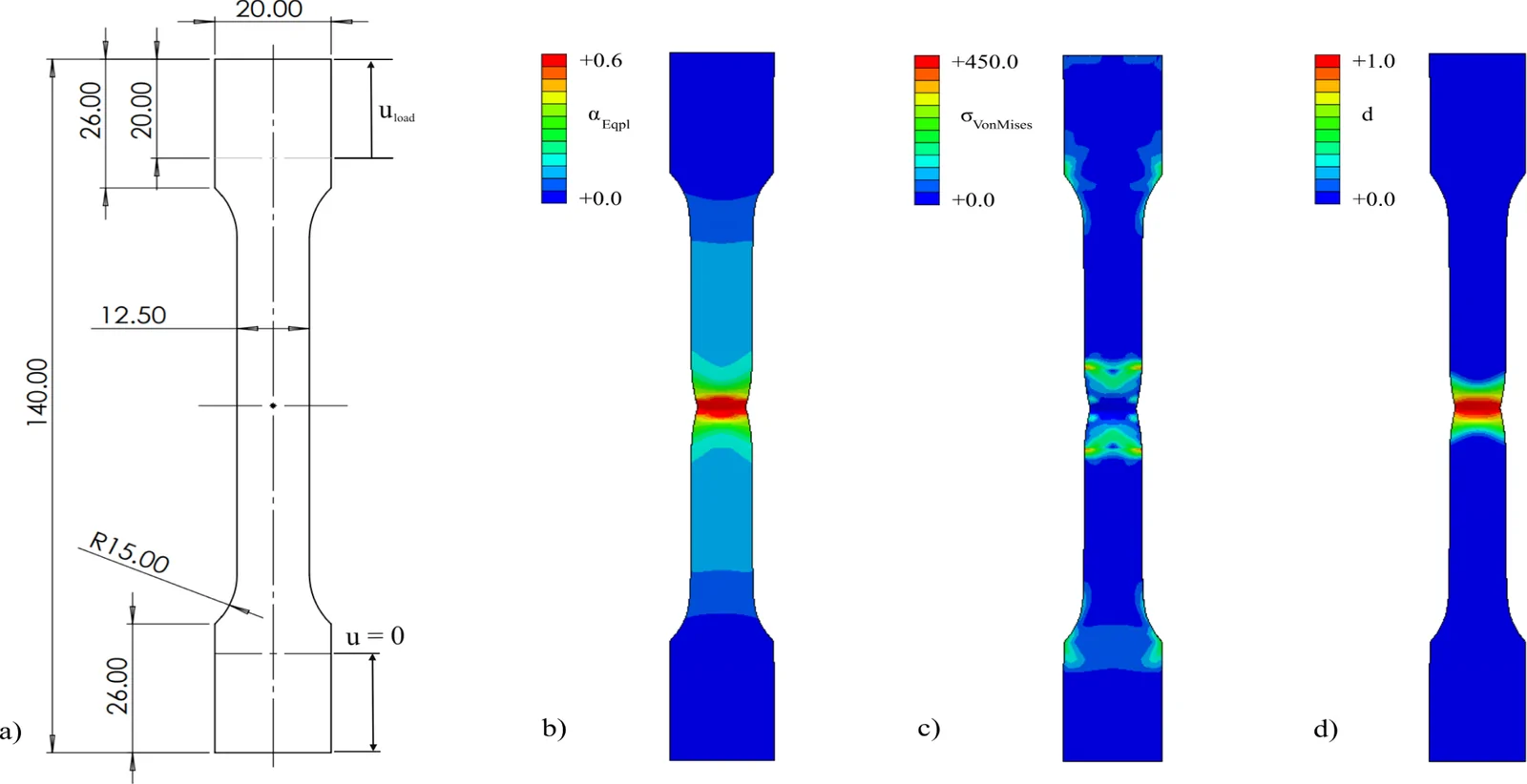
Tensile specimen geometry and contour plots of equivalent plastic strain, von Mises stress, and phase-field variable after complete fracture

In order to ensure the quality of the mathematical models, the stent stainless steel model is calibrated using experimental data provided in the work of Ambati et al. (2016). This study was selected because it applies phase-field fracture theory to finite strains in a different context, which aligns well with the nature of the present work. The technical design of the tensile specimen with a thickness of $t = 3~\text {mm}$ is shown in Fig. 7a and was chosen for this study.

**Table 1:** Material parameters for Steel-1.0553

Property	Value	[Unit]	Property	Value	[Unit]
Shear modulus, μ	73, 255	[MPa]	Saturation coefficient, δ	16.93	[–]
Bulk modulus, κ	150, 000	[MPa]	Hardening modulus, *H*	300	[MPa]
Yield stress, $\sigma _Y$	343	[MPa]	Critical fracture energy, $\psi _c$	2000	[MPa]
Ultimate tensile strength, $\sigma _\infty$	680	[MPa]	Post-critical crack driving parameter, ζ	10	[–]

As mentioned in the literature, the specimen is clamped at both the top and bottom. The bottom side is fixed to prevent any motion, while a displacement-controlled load is applied at the top until full fracture occurs. For the simulation, approximately 38, 000 hexahedral elements with eight integration points each are used. The material properties employed in the simulation are summarized in Table 1.

Figure 7b–d presents contour plots of the equivalent plastic strain, the von Mises stress, and the phase-field variable, respectively, at the final state of the tensile specimen after complete fracture. The framework employed in this work yields results in good agreement with previous studies (Ambati et al. 2016; Dittmann et al. 2018). As expected, fracture initiates in regions of highest plastic strain, leading to pronounced necking of the specimen. In the fully fractured zone, the degradation function teaches its minimum value, resulting in an near-complete loss of stiffness. Consequently, the stress levels in this region are negligible and correspond to the von Mises stress distribution shown in Fig. 7c.

**Fig. 8:**
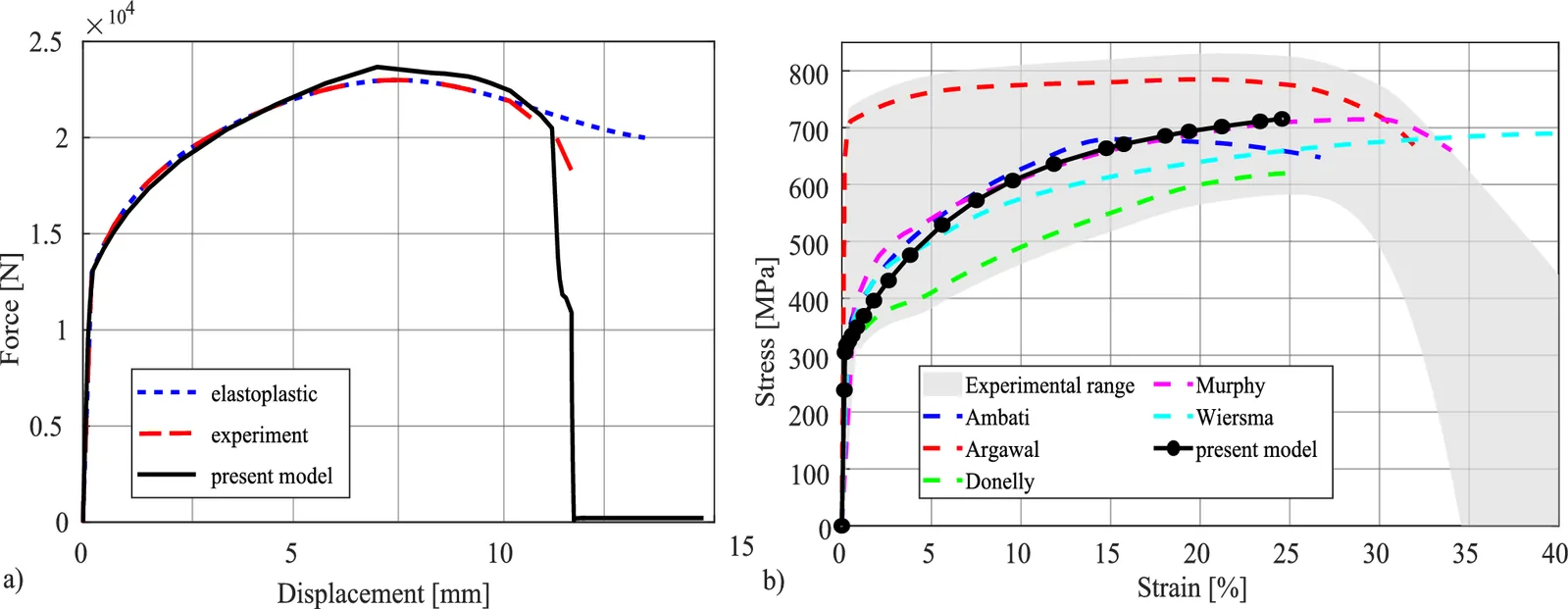
Validation of the stent material model against experimental data showing **a** the force-displacement response and **b** the stress–strain response, including comparisons with literature models

Moreover, the force-displacement diagram in Fig. 8a clearly demonstrates that the current framework adequately reproduces the elastoplastic fracture behavior and agrees well with the experimental data reported in the literature. In addition, Fig. 8b illustrates the distribution of different experimental datasets (light gray area). The studies Ambati et al. (2016); Murphy et al. (2003), and Donnelly et al. (2017) examined tensile specimens with very thin notches, comparable in size to stent struts, while Agarwal et al. (2007) and Wiersma et al. (2006) performed fatigue analyses on metallic stent struts. As can be seen, the present model, with the selected material properties, successfully replicates the behavior of several experimental test cases and remain within the shaded area.

**Table 2:** Constitutive parameters for soft tissue

Property	[Unit]	Intima	Media	Adventitia
μ	[MPa]	0.02	0.00231	0.00616
$k_1$	[MPa]	0.18259	0.00845	0.03289
$k_2$	[–]	228.58	12.84	167.31
ρ	[–]	0.4	0.3	0.3
ϕ	[$^\circ$]	13.1	24.9	75.3

**Fig. 9:**
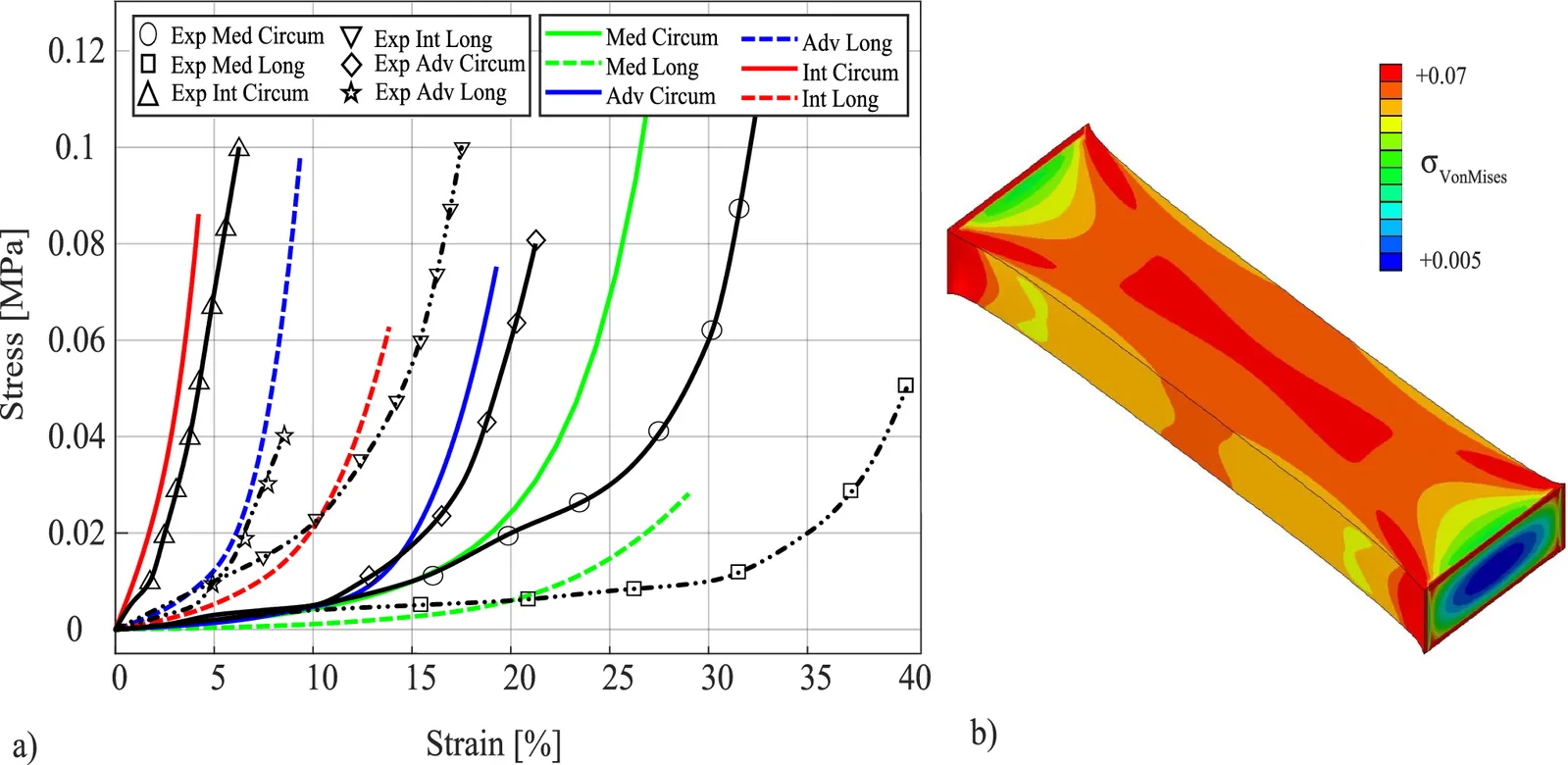
Calibration of the arterial material model showing **a** stress–strain responses under circumferential and longitudinal loading for different arterial layers and **b** the corresponding von Mises stress distribution within the arterial segment

Analogous to the previous study, the computational model for describing the behavior of the coronary arteries must be validated. For this purpose, the model is calibrated and assessed using the work (Holzapfel et al. 2005), which provides detailed experimental data and explicitly specified material parameters for all layers of coronary arteries. The experimental data were obtained from tensile tests conducted on thirteen specimens. To this end, each arterial layer was isolated, and specimen strips were carefully cut and prepared in both the circumferential and longitudinal directions. The work Holzapfel et al. (2004) provides thorough details how the samples are prepared and the experiments are conducted. The soft tissue tensile specimen adopted in this work holds the dimensions of $\text {width = 3\,mm, length = 8\,mm and thickness = 0.9 mm }$. A displacement control simulation is applied, with the specimen being meshed using approximately 20, 000 hexahedral elements (C3D8). Table 2 summarizes the constitutive parameters for each layer. As mentioned in Sec. 3, to ensure incompressibility, effect bulk modulus hold a value $\kappa \approx 10^3 \mu$ relative to the shear modulus, which results with a Poisson’s ratio of $\nu \approx 0.5$, thus guaranteeing incompressibility. In Fig. 9a, the black curves represent the experimental reference data for specimen IX from Holzapfel et al. (2005), the solid colored lines correspond to the simulation results under circumferential loading, and the dashed lines to the cases with longitudinal loading. Figure 9b depicts the von Mises stress distribution in the arterial segment, which agrees with the calibrated mechanical behavior.

As shown in Fig. 9, the proposed computational model accurately captures the mechanical response of the intima, media, and adventitia layers under longitudinal and circumferential loading. The minor deviations from the experimental data can be attributed to differences between the present model and that of Holzapfel et al. (2005), as well as to the use of an averaged arterial geometry instead of a specimen-specific configuration. Despite these differences, the overall agreement is excellent. Future work will focus on optimizing the material parameters (Alloisio and Gasser 2023; Tragoudas et al. 2024) and considering the effects of dispersed collagen fibers (Holzapfel et al. 2015).

### Crimping stents

**Fig. 10:**
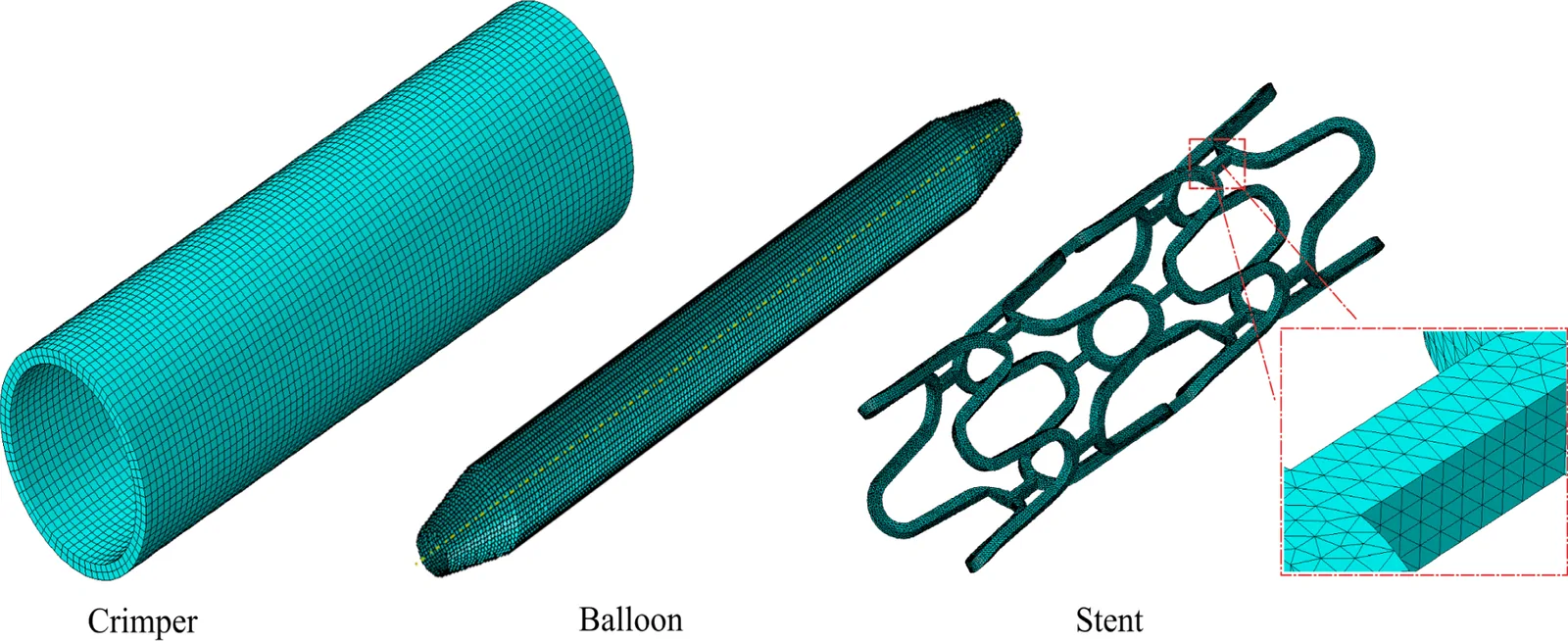
Finite element meshes of the crimping assembly components, including the crimper, balloon, and stent

As reported in the literature, coronary arteries typically have a diameter of about 3 $\text {mm}$. Stent implantation into the narrowed artery is performed non-invasively using a catheter. Studies have shown that arterial stenosis can reach up to 70% in severe cases of atherosclerosis (Achenbach et al. 2001; Meijboom et al. 2008; Kim et al. 2018; Jiangping et al. 2013). Therefore, stents must first be crimped to fit, which results in high strain levels. There have been interesting studies considering finite strain theory while using the build-in Abaqus material models (Schiavone et al. 2017), with an explicit solver whose formulation neglects convergence criteria, thereby limiting control over solution accuracy in highly nonlinear regimes (Schiavone et al. 2017; Qiu et al. 2018). However, this work not only links hyperelasticity with the phase-field implemented as UEL, thus taking into account the effect of material and geometrical nonlinearities, but also uses an implicit solver that requires a converged solution. With this motivation, crimping simulations are conducted considering different cases. As shown in Fig. 10, the simulation consists of three parts. The balloon is modeled using a Mooney-Rivlin material integrated into Abaqus. The material parameters $C_{10} = 1.03$, $C_{01} = 3.69$ and D=0 are based on literature data for polyurethane, see Schiavone and Zhao (2015). The balloon is discretized with approximately 10, 000 elements, the exact number depending on the length of the stent under investigation. Shell elements with a reduced integration scheme (S4R) are selected to accurately represent the thin balloon layer.

The crimping tool is simulated as a purely hyperelastic material using the formulation introduced in this work, neglecting the effects of plasticity and fracture. The stent is modeled using the full moldeing framework, and the calibrated material properties introduced in the previous section are used to simulate a hypothetical realistic condition. The crimper contains a similar number of elements as the balloon, but it is modeled as a solid structure with hexahedral elements with full integration (C3D8). For the stents, due to their complex geometrical design, ten-node tetrahedral elements with four Gauss points (C3D10) are employed. The thickness of the struts for all designs is set to $t=0.1 \ \text {mm}$, therefore an $\text {element size} = 0.032 \ \text {mm}$ is chosen with a length scale of $l_{c} = 0.08 \ \text {mm}$. The number of elements for the stents varies from 250, 000 up to 500, 000 depending on the design. While a finer mesh could further improve the local resolution of fracture evolution, an important distinction of the present work is that the entire stent geometry is modeled, in contrast to many existing studies that rely on symmetric boundary conditions due to idealized stent designs. Simulating the full structure is essential, as it allows global deformation effects such as bulking and rotation to develop naturally, which would otherwise be suppressed and may contribute to the initiation of phase-field damage.

The hyperelastic formulations used for all components, together with the nonlinear plasticity and fracture evolution of the stent, enable the capture of the essential deformation mechanisms during crimping process within a unified framework. Consideration of the pressure-driven contact with friction in the normal and tangential directions ensures a realistic representation of the stent-crimp interaction. Therefore, the proposed modeling approach shows very good agreement with the experimental observations reported in Wang et al. (2018).

**Fig. 11:**
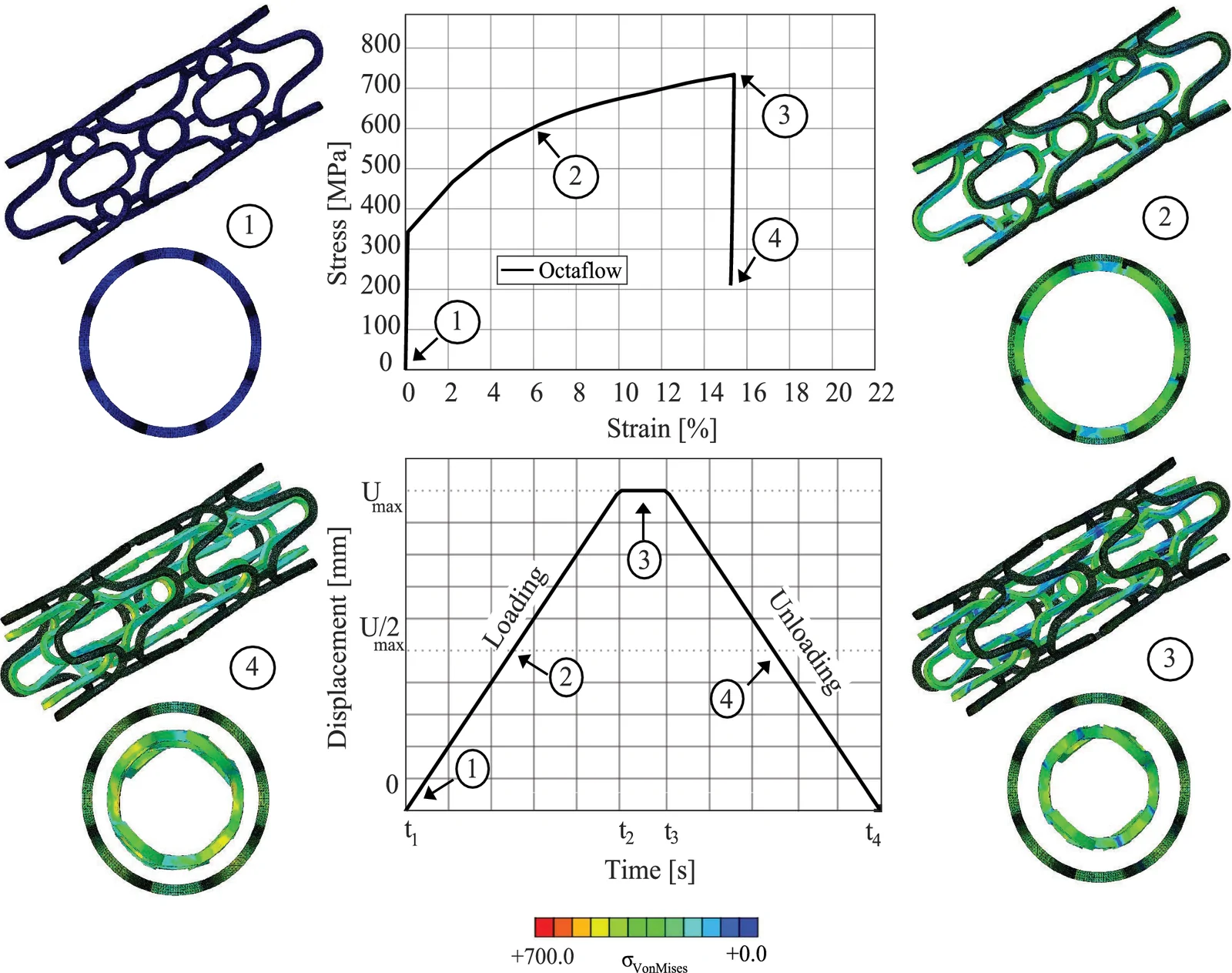
Crimp simulation of the calibrated stent, showing the stress–strain response, the loading-unloading history and the von Mises stress distributions in four selected stages

Based on published studies, the simulation is divided into three phases, as shown in Fig. 11. The first phase is the loading phase [steps (1) to (2)], in which the stent and balloon are crimped. The second phase [step (3)] is a short holding phase at maximum displacement to allow the system to reach equilibrium. The final phase [step (4)] is the unloading phase, which accounts for the influence of elastic recoil on the stent. The stress–strain curve in Fig. 11 clearly indicates that the strain values significantly exceed the threshold of 3%. According to the literature, the theory of small strains no longer accurately describes the mechanical behavior beyond this threshold, as high strain values in the range of 15% are reached in this case.

**Fig. 12:**
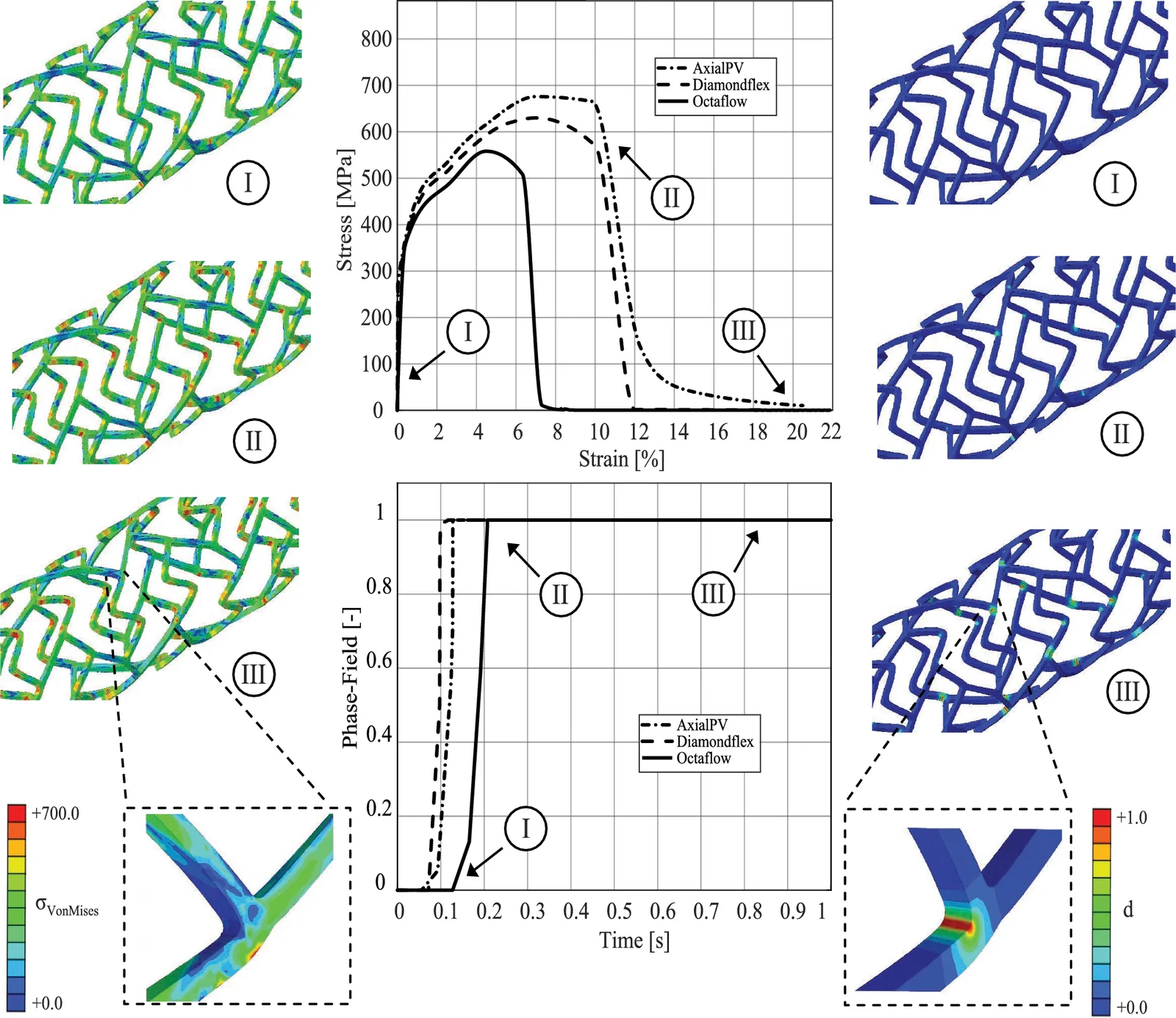
Simulation of the crimping process under extreme conditions shows the stress–strain response, the phase-field evolution, and the von Mises stress distributions for three stent designs. The contour plots correspond to the characteristic deformation stages I–III, which are represented in the stress–strain and phase-field plots

The stress–strain curve represents a crimping simulation using the calibrated model parameters, in which the effects of elasticity, nonlinear isotropic hardening, elastic recoil, and residual stresses due to plastic strain can be observed. In addition to the stress–strain curve, the contours of the von Mises stress distribution of the Octaflow design are visualized in several states. Initially [step (1)], the stent is in a stress-free state. A slow increase in load leads to deformation [step (2)] until the fully crimped state is reached [step (3)]. Subsequently, the load is removed, causing the stent diameter to increase slightly due to elastic recovery (step 4). However, due to the ongoing plastic strains, the stent exhibits permanent deformation and residual stresses. Phase-field analysis shows that the phase-field variable attains only a low value on the order of $10^{-5}$. Damage initiation at this level is typically neglected in the literature. Nevertheless, this information is not insignificant, as it indicates the onset of a potential fracture. During subsequent stent expansion and under continuous blood pulsation, which mechanically corresponds to fatigue loading, this initially low phase-field concentration generated during crimping may act as a trigger for progressive damage and potentially lead to a significant reduction in stent lifespan.

To assess the stent response under extreme conditions, scenarios representative of excessive loading, unfavorable design choices, or microstructural material irregularities are considered. Figure 12 presents the stress–strain responses of three stent designs evaluated under extreme conditions. To ensure a consistent comparison, identical material parameters, mesh resolution, and equivalent boundary and loading conditions are used for all designs. Figure 12 shows contour plots of the von Mises stress and the corresponding phase-field variables (left and right, respectively) for the DiamondFlex stent. Phase-field damage initiates in regions of maximum von Mises stress. In all cases, complete rupture occurs once the phase-field variable reaches its maximum value and the stress drops to zero.

**Fig. 13:**
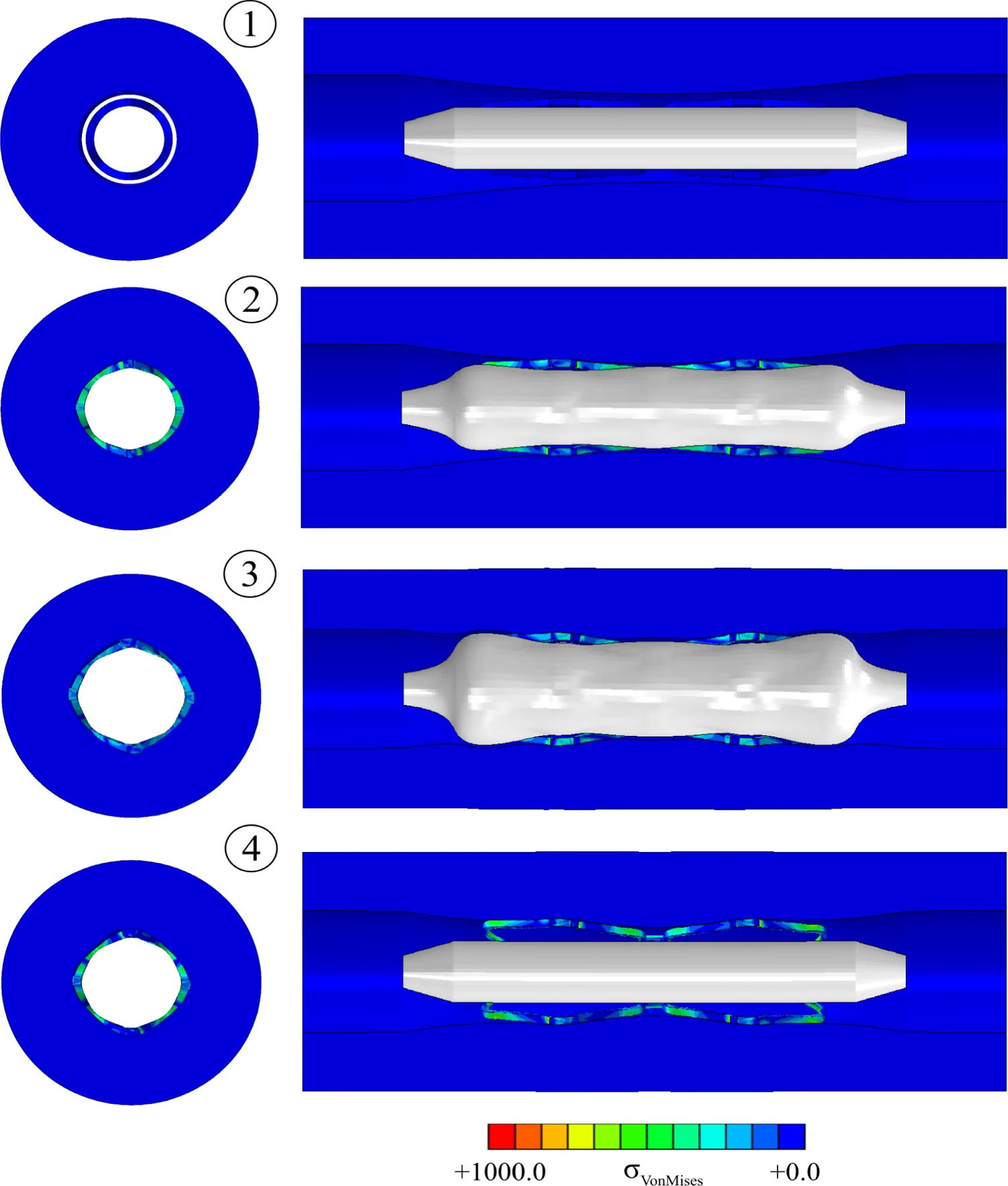
Stent deployment within a stenosed coronary artery, illustration of the arrangement of artery, stent and balloon as well as the corresponding von Mises stress distributions in four characteristic deployment stages

**Fig. 14:**
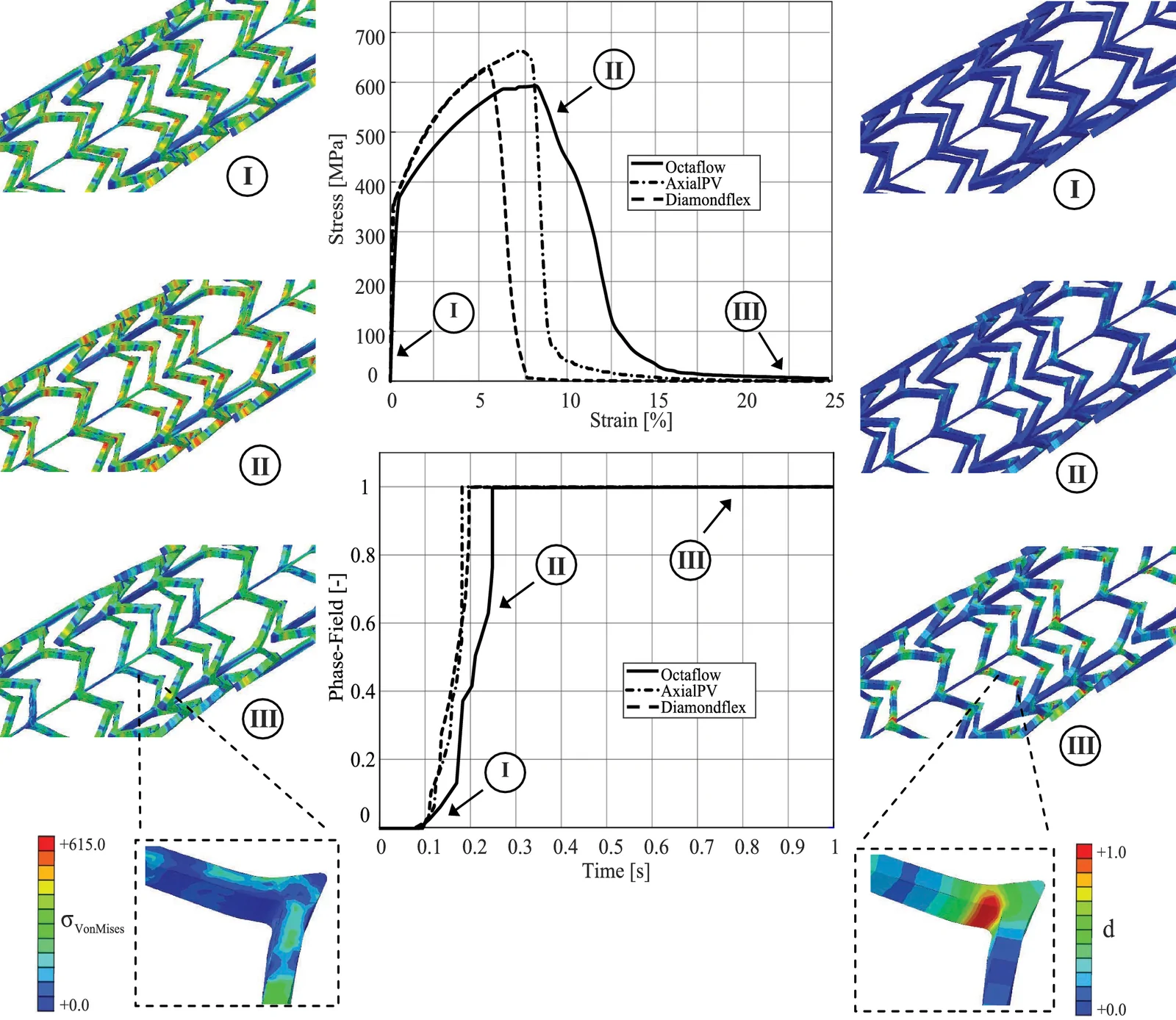
Deployment under severe loading conditions showing the stress–strain response, phase-field evolution, and von Mises stress distributions for three stent designs. Contour plots illustrate characteristic deformation stages I–III

**Fig. 15:**
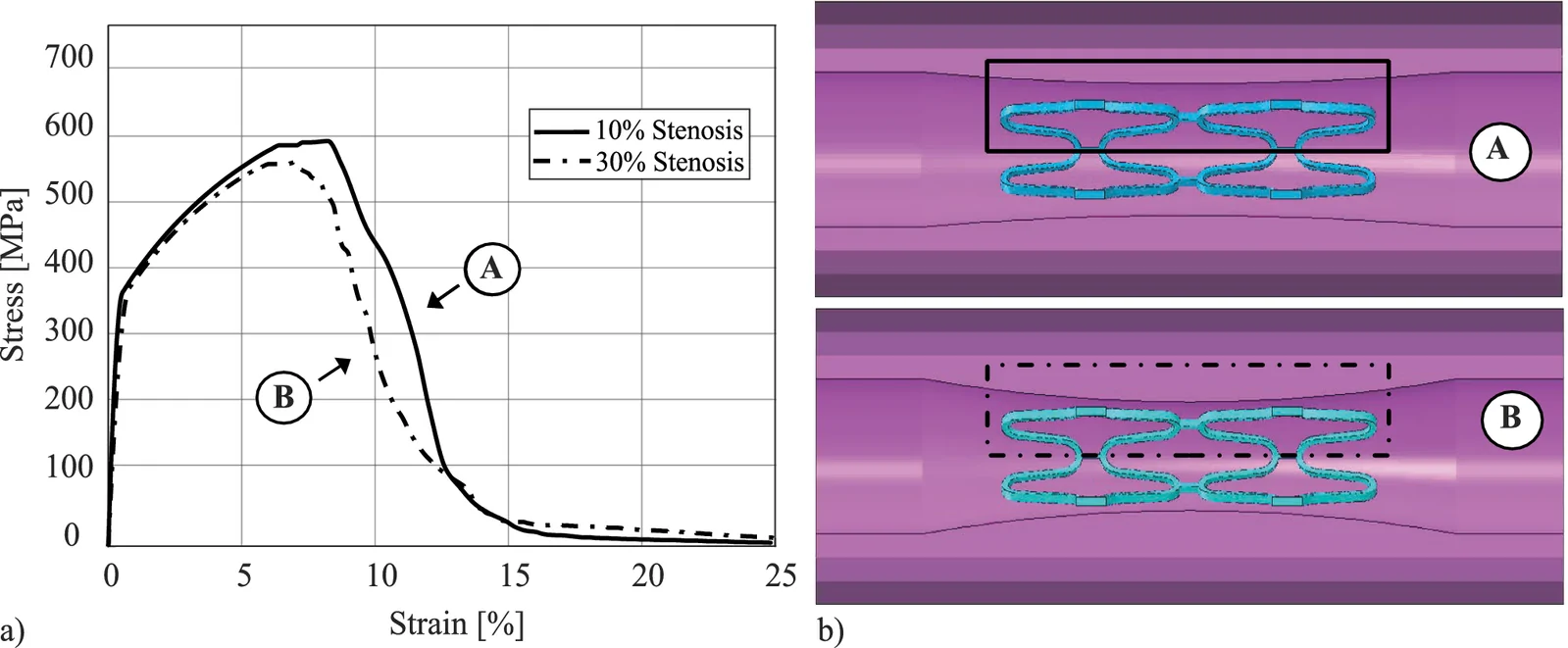
Comparison of stent deployment for 10 and 30% coronary artery stenosis, showing **a** the stress–strain response and **b** the corresponding stent-artery configurations in characteristic deployment stages (A) and (B)

### Deployment

This subsection deals with the deployment of the stent in a stenosed coronary artery, the second stage of balloon angioplasty. Figure 13 visualizes the von Mises stress distribution on the stent and the coronary artery in all phases of the deployment procedure.

At the initial stage of the deployment simulation, after positioning the crimped stent and the balloon inside the artery, all components are assumed to be in a stress-free state. Residual stresses induced during the crimping phase are not considered in this simulation, as previous studies have shown their influence to be limited. In contrast, the stresses generated after full stent deployment within the artery may have a significant impact on the structural integrity and lifetime of the stent. In this simulation, an air pressure of approximately $1.0~\text {MPa}$ is applied to the balloon, and its inflation leads to the expansion of the stent, as illustrated in steps (2) and (3), resulting in the widening of the narrowed coronary artery.

The applied air pressure may vary depending on the stent design, degree of stenosis, and geometric characteristics of the artery. In practice, it has been shown that the application of air pressure is repeated in some cases to ensure complete stent expansion. Once full pressure is applied and the stent has reached its maximum expansion, as shown in Fig. 13, the pressure is held constant for a short time, as illustrated in Fig. 11 for the loading-unloading history curve. The balloon is then deflated until it returns to its original state and is subsequently withdrawn together with the catheter. During deflation, the stent recoils elastically and reaches its final deformed configuration [step (4)] determined by the accumulated plastic strain, while the coronary artery remains dilated. In this simulation, the coronary artery is modeled as a three-layer composite structure with two families of fibers in each layer, using the material parameters introduced in the previous section. For simplicity, in the non-atherosclerotic regions each arterial layer is assumed to have a thickness of $0.3~\text {mm}$. It is noted that layer thickness ratios may vary between patients as reported in Holzapfel et al. (2005), hence an average ratio is adopted in this work. An early-stage atherosclerotic configuration is considered in this study. Hence, the plaque is assumed to be confined within the intima, which is locally thickened. The plaque is therefore assigned material properties consistent with those of the intima, following common assumptions in the literature. Analogous to the crimping simulation, under realistic operating conditions the stent shows no significant damage during deployment.

To demonstrate the robustness of the proposed framework, the deployment procedure under high loading conditions, similar to the crimping case, is investigated for three different stent designs, as shown in Fig. 14. All stents are initially crimped to approximately 60% of their original diameter using displacement-controlled loading, without explicitly modeling the crimper or balloon. This approach ensures consistent and comparable conditions for the different stent designs while simultaneously subjecting the model to large deformations. The corresponding von Mises stress distributions and the phase-field evolution in the characteristic stages I–III are shown in Fig. 14. The DiamondFlex and AxialPV designs share similar geometric features, such as length, diameter, and strut thickness, as they were developed by the authors. Consequently, they exhibit a similar stress–strain response, as shown in Fig. 14. Although damage initiation occurs at approximately the same stage for both designs, the strain level at which complete rupture occurs differs. This underscores the importance of the design features. In contrast, the OctaFlow design, despite its simpler geometric pattern, exhibits higher resistance and withstands greater strains before its stiffness drops to zero.

In addition to the loading conditions and stent design, the degree of coronary stenosis at which balloon angioplasty with stent implantation is performed also plays an important role. Since arterial geometry and the degree of stenosis vary between patients, their influence on stent response is examined here, as illustrated in Fig. 15. In this study, the same simulation setup is employed for all cases. Thus, material properties as well as boundary and loading conditions are kept identical. The only variation considered is the geometry of the coronary artery. As described, cases with 10 and 30% stenosis were examined. The results indicate that a higher degree of stenosis can lead to earlier stent rupture under extreme conditions. This occurs because the stent comes into contact with the arterial wall earlier during expansion, which limits its deformation. The resulting reaction forces promote a more rapid propagation of phase-field damage.

## Conclusions and future aspects

This work presents a computational framework based on finite-strain phase-field formulations to investigate the mechanical response and failure of coronary stents during crimping and deployment in contact with anisotropic hyperelastic arterial tissue. The simulations demonstrated how crimping-induced stresses and stent-artery interaction triggered the initiation and evolution of stent fractures, resulting in stress redistribution, inelastic deformation, and partial elastic recoil after balloon deflation. The results highlighted that both stent geometry and arterial material properties play a crucial role in fracture behavior and overall mechanical stability.

In its current form, the study focuses on the core mechanical mechanisms and does not explicitly incorporate additional factors influencing the in vivo behavior of coronary arteries. Soft tissues, however, exist in a stable mechanical state referred to as homeostasis, which can be represented by incorporating preloading effects. Previous studies (Grytsan et al. 2017; Gierig et al. 2024) have proposed computational approaches that capture both pre-stretch and homeostatic conditions, leading to the development of arterial residual stresses. Extending the present formulation in this direction will enable a more consistent representation of in vivo behavior. Furthermore, we intend to incorporate more realistic coronary layered models that consider the fibrous cap, lipids, and calcified plaque. Additional developments will address arterial remodeling due to damage induced during deployment (Noble et al. 2016; Deo et al. 2026), incorporating fiber dispersion models and optimization techniques for material parameter calibration. Future efforts will target fatigue failure (Kalina et al. 2023), biodegradable materials, and corrosion effects (Baktheer et al. 2026; Gopakumar et al. 2026) in stents. The framework will also be integrated with physics-based machine learning (ϕML) for rapid evaluation (Aldakheel et al. 2025; Elsayed et al. 2026; Baktheer and Aldakheel 2025) and design optimization. Finally, drug transport governed by Fick’s law will be incorporated to model drug-eluting stents and mitigate restenosis.

These advances aim to enable the realization of a new class of cardiovascular implants, referred to here as smart stents, which combine mechanical reliability, digital intelligence, and continuous patient monitoring. While preliminary studies explored the integration of sensors into stent structures, these efforts primarily focused on electronic components and data transmission (Jeong et al. 2023; Park et al. 2016; Antonini et al. 2025). In contrast, the concept proposed here emphasizes the interaction between material behavior and the structural response of the implant, which has received little attention to date (Shin et al. 2022; Hoare et al. 2019).

## Data Availability

No datasets were generated or analysed during the current study.
